# Mitochondrial carrier homolog 2 is important for mitochondrial functionality and non-small cell lung cancer cell growth

**DOI:** 10.1038/s41419-025-07419-0

**Published:** 2025-02-13

**Authors:** Yong Zhao, Siyang Wu, Guohong Cao, Peidong Song, Chang-gong Lan, Lin Zhang, Yong-hua Sang

**Affiliations:** 1 Department of Thoracic Surgery, Affiliated Hospital of Jiangnan University, Wuxi, China; 2 Respiratory Intensive Care Unit, Affiliated Hospital of YouJiang Medical University for Nationalities, Baise, China; 3 Department of Respiratory and Critical Care Medicine, Binzhou Medical University Hospital, Binzhou Medical University, Binzhou, China; 4 Department of Cardiothoracic Surgery, the Second Affiliated Hospital of Soochow University, Suzhou, China; 5 Guangxi Key Laboratory of basic and translational research of Bone and Joint Degenerative Diseases, Guangxi Biomedical Materials Engineering Research Center for Bone and Joint Degenerative Diseases, Baise, China; 6 Department of Orthopaedics, Affiliated Hospital of Youjiang Medical University for Nationalities, Baise, China; 7 Department of Thoracic Surgery, Suzhou Ninth People’s Hospital Affiliated to Soochow University, Suzhou, China

**Keywords:** Non-small-cell lung cancer, Targeted therapies

## Abstract

Discovering new molecular targets for non-small cell lung cancer (NSCLC) is critically important. Enhanced mitochondrial function can promote NSCLC progression by enabling metabolic reprogramming, resistance to apoptosis, and increased cell proliferation. Mitochondrial carrier homolog 2 (MTCH2), located in the outer mitochondrial membrane, is pivotal in regulating mitochondrial activities. This study examines MTCH2 expression and its functional role in NSCLC. Bioinformatic analysis showed that MTCH2 is overexpressed in NSCLC tissues, correlating with poor prognosis and other key clinical parameters of the patients. In addition, single-cell sequencing data revealed higher MTCH2 expression levels in cancer cells of NSCLC tumor mass. Moreover, MTCH2 is also upregulated in locally-treated NSCLC tissues and multiple primary/established human NSCLC cells. In various NSCLC cells, silencing MTCH2 via targeted shRNA or knockout (KO) using the CRISPR/Cas9 method significantly hindered cell proliferation, migration and invasion, while inducing apoptosis. MTCH2 knockdown or KO robustly impaired mitochondrial function, as indicated by reduced mitochondrial respiration, decreased complex I activity, lower ATP levels, lower mitochondrial membrane potential (mitochondrial depolarization), and increased reactive oxygen species (ROS) production. Conversely, ectopic overexpression of MTCH2 in primary NSCLC cells enhanced mitochondrial complex I activity and ATP production, promoting cell proliferation and migration. In vivo, the intratumoral injection of MTCH2 shRNA adeno-associated virus (aav) impeded the growth of subcutaneous xenografts of primary NSCLC cells in nude mice. In MTCH2 shRNA aav-injected NSCLC xenograft tissues, there was decreases in MTCH2 expression, mitochondrial complex I activity, ATP content, and the glutathione (GSH)/glutathione disulfide (GSSG) ratio, but increase in thiobarbituric acid reactive substances (TBAR) activity. Additionally, MTCH2 silencing led to reduced Ki-67 staining but increased apoptosis in NSCLC xenografts. Collectively, these findings demonstrate that overexpressed MTCH2 promotes NSCLC cell growth potentially through the maintenance of mitochondrial hyper-function, highlighting MTCH2 as a novel and promising therapeutic target for treating this disease.

## Introduction

Lung cancer imposes a profound socioeconomic burden worldwide, resulting in a considerable number of annual cancer-related deaths [1–3]. In 2019 alone, the United States reported over 220,000 new lung cancer cases and 140,000 related fatalities [1, 2]. The disease manifests in two primary histological forms, including small cell lung cancer (SCLC) and non-small cell lung cancer (NSCLC) [1, 2]. NSCLC is particularly prevalent, accounting for 85% of all lung cancer cases, with adenocarcinoma (LUAD) and squamous cell carcinoma (LUSC) two most frequently diagnosed subtypes [1–4]. The treatment of NSCLC requires a comprehensive, multidisciplinary approach that includes surgery, chemotherapy, immunotherapy, and targeted therapies [5, 6]. Regrettably, a significant number of NSCLC patients are diagnosed at advanced stages or suffer a relapse even after undergoing surgery with curative intent, resulting in poor prognosis [7, 8].

Genes with abnormal expression or mutations, along with disrupted signaling pathways, play a crucial role in the onset, progression, and treatment resistance of NSCLC [9–13]. Current targeted therapies include reagents aimed at EGFR mutations (such as gefitinib, and osimertinib), ALK rearrangements (such as crizotinib and alectinib), BRAF mutations (such as dabrafenib and trametinib), and MET alterations (such as crizotinib and capmatinib) [9, 14–16]. Despite these treatments, the prognosis for patients with advanced NSCLC remains poor [9, 14, 17, 18]. Identifying new molecular targets essential for NSCLC cell progression is critical for developing better therapeutic approaches [9–13].

Mitochondria play a crucial role in oxidative phosphorylation (OXPHOS), the production of ATP, and amino acid metabolism [19–23]. They are also essential for the synthesis of macromolecules, the oxidation of fatty acids, and maintaining ion balance [19–23]. Additionally, mitochondria serve as central hubs for signal transduction and the regulation of apoptosis [19–23]. Mitochondrial hyper-function will support NSCLC cell growth by enhancing ATP production, biosynthetic pathways, and redox homeostasis, providing essential energy for rapid proliferation of cancer cells [24–27]. Elevated mitochondrial activity increases tricarboxylic acid cycle intermediates, facilitating nucleotide, amino acid, and lipid synthesis. Additionally, moderate levels of reactive oxygen species (ROS) generated by hyperactive mitochondria activate oncogenic pathways, promoting cell survival and proliferation [28, 29].

The solute carrier 25 family protein mitochondrial carrier homolog 2 (MTCH2) deviates from its usual function of transporting substrates across the inner mitochondrial membrane (IMM) by predominantly positioning itself on the outer mitochondrial membrane (OMM) [30, 31]. At this location, MTCH2 operates as an insertase, embedding cytoplasmic α-helical proteins into the OMM [30, 31]. Additionally, it acts as a scramblase, aiding in the movement of phospholipids between the layers of the mitochondrial membrane, vital in maintaining mitochondrial dynamics and functionality [30, 31]. MTCH2 can also associate with pro-apoptotic proteins including truncated BID, aiding their movement to the mitochondria, which in turn regulates the permeability of the outer mitochondrial membrane and triggers apoptosis [32]. Studies highlight MTCH2’s crucial role across multiple biological and pathological contexts, impacting metabolic syndromes, neurodegeneration, embryogenesis, and reproductive health [31]. Recent research suggests that MTCH2 may play an oncogenic role in human cancers [33–35]. This study investigates its expression and possible functional role in NSCLC.

## Materials and methods

### Reagents and antibodies

Puromycin, cell-culturing medium, FBS, Matrigel, z-VAD-fmk, z-DEVD-fmk, polybrene, protein and RNA assay reagents, the Cell Counting Kit-8 (CCK-8) were obtained from Sigma-Aldrich (St. Louis, MO). Fluorescent dyes, including EdU (5-ethynyl-2’-deoxyuridine), TUNEL (terminal deoxynucleotidyl transferase dUTP nick end labeling), CellROX, DCF-DA, and JC-1, were acquired from Invitrogen Thermo-Fisher (Suzhou, China). All antibodies employed in the experiments were purchased from Cell Signaling Technology (Danvers, MA, USA) and Abcam (Shanghai, China). Viral constructs, sequences, and primers were provided by Genechem (Shanghai, China) unless otherwise specified.

### Cells

A549 NSCLC cell line was sourced from the American Type Culture Collection and grown in high-glucose DMEM with 10% fetal bovine serum (FBS). Primary human NSCLC cells (“pNSCLC-1”, “pNSCLC-2”, and “pNSCLC-3”) from three different patients, and primary human lung epithelial cells from two patients (“pEpi1” and “pEpi2”), were described in prior studies [26, 36, 37]. Routine assays for mycoplasma, microbial impurities, STR profiling, population doubling, and cellular morphology were performed. The Soochow University Ethics Committee authorized the use of primary human cells, adhering to the Declaration of Helsinki guidelines.

### Human tissues

This study included a group of 20 patients with stage III-IV LUAD, aged 41 to 85 years, treated at the authors’ institutions. Each participant gave written consent prior to inclusion. Fresh tumor samples and nearby healthy lung tissues were gathered during surgery and rapidly stored in liquid nitrogen for further study. The management of these samples followed protocols sanctioned by the Soochow University Ethics Committee, aligning with the Declaration of Helsinki standards.

### Western blotting

Tissues and cells were lysed by radioimmunoprecipitation assay (RIPA) buffer enhanced with protease inhibitors (Beyotime, Wuxi, China). Protein concentration was measured using a bicinchoninic acid assay kit (Invitrogen, Suzhou, China). Proteins were then resolved on SDS-PAGE (sodium dodecyl sulfate–polyacrylamide gel electrophoresis) and transferred to polyvinylidene difluoride (PVDF) membranes (EMD Millipore, Darmstadt, Germany). After blocking, the membranes were incubated overnight with primary antibodies, followed by a 45 min incubation with secondary antibodies. Target protein bands were visualized using the enhanced chemiluminescence (ECL) system. A Sigma-Aldrich kit was used to isolate mitochondria following the provided protocol. In brief, NSCLC cells were homogenized to disrupt membranes while keeping organelles intact. The resulting homogenate underwent differential centrifugation to remove larger debris and isolate the mitochondrial fraction. The uncropped blotting images are listed in Supplementary Fig. S1.

### Quantitative real-time polymerase chain reaction (qRT-PCR)

Total RNA was extracted from cells and tissues via TRIzol reagents (Takara, Otsu, Japan). RNA concentration was measured, and reverse transcription to cDNA was conducted with a Reverse Transcription Kit (Applied Biosystems, Foster City, CA). The qRT-PCR assays were performed with the SYBR Green real-time PCR Kit (Takara) on the Bio-Rad system (Bio-Rad, Hercules, CA). mRNA levels were quantified using the 2^−ΔΔCt^ method, with *glyceraldehyde-3-phosphate dehydrogenase* (*GAPDH*) serving as the internal control. The mRNA primers for *MTCH1* and *GAPDH* were reported previously [38], and *MTCH2* mRNA primers described in an early study [33].

### MTCH2 shRNA

To silence MTCH2, shRNA experiments were conducted on NSCLC cells or lung epithelial cells grown in complete medium with polybrene at 50% confluence. Lentiviral particles carrying MTCH2 shRNA in the GV369 construct, obtained from Genechem (Shanghai, China), were added to the cells at multiplicity of infection (MOI) of 12.5. Following a 60 h infection period, the cells were treated with puromycin-containing complete medium to select for stable colonies for six passages. Two specific shRNAs with non-overlapping sequences were employed: shMTCH2-S1 and shMTCH2-S2. Control cells were infected with lentiviral particles containing a scramble control shRNA (shC) [26, 36, 37]. *MTCH2* mRNA and protein expression levels in stable cells were monitored regularly. For in vivo experiments, the shMTCH2-S1 sequence or the shC sequence were cloned into an adeno-associated virus (aav) construct as previously described [26, 36], and viral particles were subsequently generated.

### CRISPR/Cas9-mediated MTCH2 knockout (KO)

Gene editing experiments for *MTCH2* were performed in primary NSCLC cells cultured in complete medium with polybrene at 50% confluence. Cells were first infected with a Cas9-expressing lentivirus [26, 36, 37], and stable cells were selected. These cells were then transduced with a lentivirus carrying a CRISPR/Cas9-MTCH2-KO puro-construct with MTCH2-specific sgRNA, again provided by Genechem (Shanghai, China). Following puromycin selection, stable cells were isolated and distributed into 96-well plates at single-cell density. Sequencing around the targeted MTCH2 site confirmed gene disruption, and KO was verified by Western blotting. Two stable single-cell-derived MTCH2 KO selections (koMTCH2-Slc1 and koMTCH2-Slc2) were established, all showing depleted MTCH2 protein levels. Control cells were infected with a lentivirus containing a CRISPR/Cas9-empty control vector (koC) as described previously [26, 36].

### MTCH2 overexpression

To overexpress MTCH2, NSCLC cells were infected with a lentivirus carrying the MTCH2-expressing construct (GV369) at MOI of 10. This construct included the MTCH2 cDNA sequence without a tag, and the infection lasted for 60 h. Following this, cells were cultured in puromycin-containing complete medium to establish stable cells after six passages. The expression levels of *MTCH2* mRNA and protein in these stable cells were regularly monitored.

### EdU staining

As reported early [26, 36, 37], NSCLC cells or lung epithelial cells were cultured in 12-well plates and allowed to grow for indicated periods. The cells were then fixed with paraformaldehyde and permeabilized with Triton X-100 to allow dye penetration. EdU and DAPI dyes were then applied to stain the cells. Fluorescence microscopy (Nikon, Minato-ku, Tokyo) was employed to capture images of the stained cells. The ratio of EdU-positive cells to DAPI-stained nuclei was determined by analyzing five randomly selected fields of view.

### In vitro cell migration and invasion assays

As reported early [26, 36, 37], cells were seeded onto the upper surfaces of “Transwell” inserts (Fisher-Scientific, Waltham, MA) and allowed to migrate for 24 h. The cells that migrated to the lower surface of the inserts were then fixed and stained using crystal violet. For invasion assays, the “Transwell” inserts were pre-coated with Matrigel (100 µg/mL, Sigma).

### TUNEL staining

NSCLC cells or lung epithelial cells were seeded into 96-well plates and cultured for predetermined time periods. Cells were co-stained with DAPI and TUNEL reagents. The TUNEL-positive nuclei were visualized using a fluorescence microscope (Nikon). The percentage of TUNEL-positive cells relative to the total number of DAPI-stained nuclei was determined by analyzing five randomly selected fields of view.

### Flow cytometry

NSCLC cells with the designated genetic treatments were cultured for designated time periods. After harvesting, cells at a concentration of 1 ×10^6^ cells/mL were washed and resuspended in 1× binding buffer (containing 10 mM HEPES, 140 mM NaCl, and 2.5 mM CaCl_2_ at pH 7.4) containing Annexin V-FITC (1 µg/mL) and PI (5 µg/mL). The cell populations were analyzed using a Beckman Coulter CytoFLEX flow cytometer (Brea, CA). For cell cycle analyses, PI dye was only added.

### Other fluorescence dye assays

As described [39], after specified incubation periods, NSCLC cells on cover-slides were supplemented with fluorescent dyes including CellROX, JC-1, and DCF-DA. The cell slides were then washed with cold PBS and fluorescence images were taken using a Zeiss Axio Observer microscope, with the relative fluorescence intensity measured and quantified via the ImageJ software.

### Mitochondrial complex I activity and ATP assays

Mitochondrial complex I activity in cell and tissue lysates was assessed via a Sigma colorimetric kit, which spectrophotometrically measures the conversion of NADH to NAD + . The reduction in absorbance at 360 nm indicated the relative complex I activity. ATP concentrations in cellular and tissue lysates were measured using a Sigma colorimetric kit following standard protocols. For each sample, 25 μL of lysates containing 25 μg of total proteins were used for analysis.

### Oxygen consumption rate (OCR)

OCR was evaluated using a XF24 Extracellular Flux Analyzer (Agilent Seahorse Bioscience) according to established procedures [40]. Cells were sequentially exposed to 1 μM oligomycin, 0.5 μM FCCP (carbonyl cyanide-p-trifluoromethoxyphenylhydrazone), and a combination of 0.5 μM antimycin A and rotenone to measure basal, ATP-linked, maximal, and non-mitochondrial OCR [40]. Results were normalized to intracellular protein concentrations.

### Thiobarbituric acid reactive substance (TBAR) assay of lipid peroxidation

Tissue lysates, each containing 35 μg proteins per sample, were analyzed using a TBAR kit from Cayman Chemical (Ann Arbor, MI). This kit specifically quantifies lipid peroxidation and malondialdehyde (MDA) content through a colorimetric method. TBAR signal intensity was measured at 535 nm, using 600 nm as the reference.

Other cellular functional studies, including CCK-8 assay of cell viability, medium lactate dehydrogenase (LDH) assay of cell death, colony formation assay, Caspase-3/Caspase-9 activity assay, glutathione (GSH)/glutathione disulfide (GSSG) ratio measurement, and cytosol cytochrome C ELISA assay were described in detail in other studies [26, 36, 37, 39].

### Xenograft studies

Nude mice, including equal numbers of males and females aged 6–8 weeks and weighing between 17.9–18.4 g, were obtained from the Animal Center at Soochow University and housed under standard conditions. To create pNSCLC-1 xenografts, six million cells were injected subcutaneously into the flanks of each mouse. Tumors developed within three weeks, reaching approximately 100 mm³ in volume. The mice were then divided into two groups: one received intratumoral injections of adeno-associated virus(aav)-packed MTCH2 shRNA, and the other received aav-packed scramble control shRNA. Virus injections were administered twice, 72 h apart [26, 36]. All animal procedures were approved by the Institutional Animal Care and Use Committee (IACUC) and Soochow University Animal Ethics Review Board.

### Immunohistochemistry and tissue fluorescence staining

Xenograft tumors were preserved and embedded in paraffin. These samples were then cut into 4 μm thick sections. To prepare for staining, sections were deparaffinized with xylene and rehydrated through a series of graded alcohol solutions. Antigen retrieval was performed by heating in a citrate buffer. To prevent non-specific binding, blocking was applied before incubating the slides with a primary antibody. Following incubation, the slides were were subjected to IHC staining of nuclear Ki-67 using a commercial Ki-67 staining kit from Biyuntian (Wuxi, China). Alternatively, TUNEL staining was conducted in xenograft section via a kit from Biyuntian, followed by washing and counterstaining with DAPI to highlight the cell nuclei. The sections were then mounted with an anti-fade medium and examined under a fluorescence microscope.

### Statistical analyses

Statistical analyses were conducted with investigators blinded to group allocation in all in vitro experiments. Normally distributed numeric data were presented as mean ± SD (standard deviation). Differences among multiple groups were analyzed using one-way analysis of variance (ANOVA), followed by Dunnett’s test with SPSS 23.0 (Chicago, CA). For comparisons between two groups, Student’s *t* test was utilized. A significance level of *P* < 0.05 was considered statistically significant. Log-Rank Test was employed for calculating *P* values for Kaplan–Meier survival curve analyses. All in vitro experiments were performed five times, yielding consistent results.

## Results

### Bioinformatical studies show *MTCH2* overexpression in NSCLC tissues

**Fig. 1:**
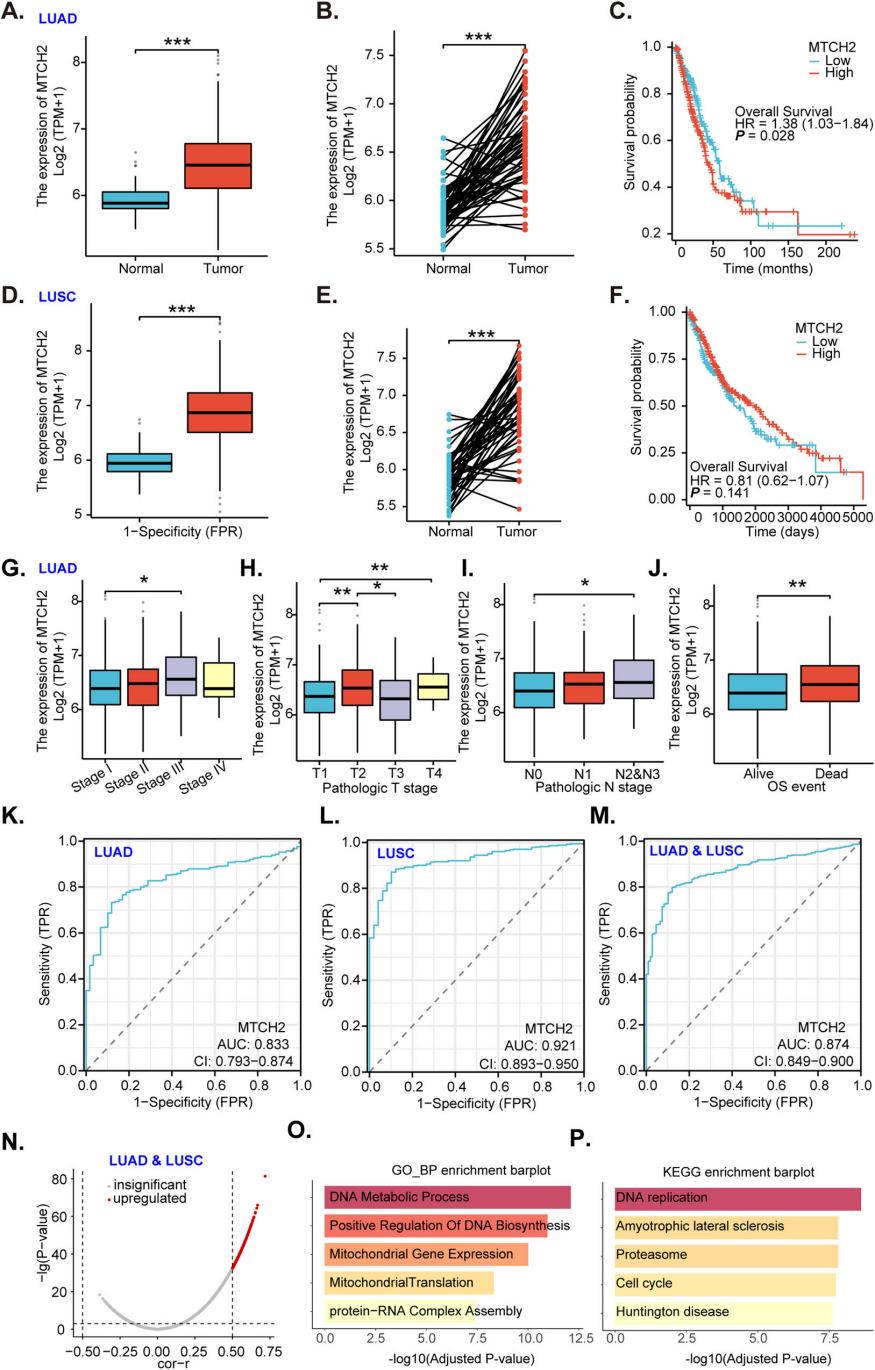
Bioinformatical studies show *MTCH2* overexpression in NSCLC tissues. The Cancer Genome Atlas Lung adenocarcinoma (TCGA-LUAD) dataset shows *MTCH2* mRNA transcript levels in described LUAD tissues (“Tumor”) and normal lung tissues (“Normal”) (**A**, **B**, **G**–**J**). Kaplan–Meier survival curves showing the relationship between *MTCH2* expression and LUAD patients’ overall survival (**C**). The Cancer Genome Atlas lung squamous cell carcinoma (LUAD-LUSC) dataset shows *MTCH2* mRNA transcript levels in described LUSC tissues (“Tumor”) and normal lung tissues (“Normal”) (**D** and **E**). Kaplan–Meier survival curves showing the relationship between *MTCH2* expression and LUSC patients’ overall survival (**F**). The receiver operating characteristic (ROC) curves assessing *MTCH2* expression for its predictive value in LUAD patients (**K**), LUSC patients (**L**) and combined (LUAD & LUSC) patients (**M**) are presented. Identification of 348 co-expressed genes (CEGs) correlated with *MTCH2* in the TCGA-LUAD and TCGA-LUSC datasets (*P* < 0.05, *R* > 0.5) (**N**). GO (**O**) and KEGG (**P**) enrichment analysis of the identified CEGs, showing associations with processes and signaling cascades. “TPM” stands for transcripts per million. “AUC” stands for area under the curve. “CI” stands for confidence interval. “HR” stands for hazard ratio. “TPR” stands for true positive rate. “FPR” stands for false positive rate. “OS” stands for overall survival. * indicates *P* < 0.05. ** indicates *P* < 0.01. *** indicates *P* < 0.001. Student’s *t* test for **A**, **B**, **D**, **E** and **J**; Log-Rank Test for **C** and **F**; ANOVA with Dunnett’s test for **G**–**I**.

We began our investigation by accessing the The Cancer Genome Atlas Lung adenocarcinoma (TCGA-LUAD) database to gather *MTCH2* expression data. Our analysis revealed an increased presence of *MTCH2* mRNA transcripts in LUAD tissues (“Tumor”) compared to normal lung tissues (“Normal”) (Fig. 1A). Paired sample analysis confirmed higher *MTCH2* expression in LUAD tissues versus their matched normal counterparts (Fig. 1B). Kaplan–Meier survival curves indicated that elevated *MTCH2* levels in LUAD are associated with poorer overall patient survival (Fig. 1C). Furthermore, data from The Cancer Genome Atlas for lung squamous cell carcinoma (TCGA-LUSC) indicated increased *MTCH2* mRNA levels in LUAD tissues (Fig. 1D). *MTCH2* expression was also significantly higher in LUSC tissues compared to paired normal counterparts (Fig. 1E). However, there was no significant difference in overall survival between patients with low- and high-*MTCH2* levels in LUSC (Fig. 1F). In LUAD tissues, *MTCH2* expression was elevated in cases with higher pathological stages (Fig. 1G, H). *MTCH2* levels are also significantly higher in LUAD tissues with N2/N3 lymph node metastasis compared to those with N0 or N1 lymph node metastasis (Fig. 1I). Additionally, *MTCH2* is overexpressed in LUAD tissues of deceased patients (“Dead”) compared to those of living patients (“Alive”) (Fig. 1J).

Receiver Operating Characteristic (ROC) curve analysis was thereafter performed using *MTCH2* expression data from TCGA-LUAD and TCGA-LUSC. The results showed an area under the curve (AUC) of 0.833 for LUAD (Fig. 1K) and 0.921 for LUSC (Fig. 1L), suggesting that *MTCH2* overexpression could be a viable diagnostic marker for LUAD and LUSC. Additionally, when combining *MTCH2* expression data for LUAD plus LUSC, the ROC analysis yielded an AUC of 0.874 (Fig. 1M), further supporting the potential of *MTCH2* overexpression as a diagnostic marker for NSCLC. In the TCGA-LUAD and TCGA-LUSC datasets, *MTCH2* correlation gene analysis was performed with thresholds set at *P* < 0.05 and a correlation coefficient *R* > 0.5. This analysis identified 348 co-expressed genes (CEGs) (Fig. 1N). Subsequent Gene Ontology (GO) (Fig. 1O) and Kyoto Encyclopedia of Genes and Genomes (KEGG) enrichment (Fig. 1P) analyses of these genes revealed significant associations with various biological processes, including DNA metabolic processes, DNA biosynthesis, cell cycle regulation, and mitochondrial gene expression. These findings suggest that MTCH2 may play a critical role in these essential cellular function of NSCLC.

### Single cell sequence data shows *MTCH2* overexpression in cancer cell population of NSCLC mass

**Fig. 2:**
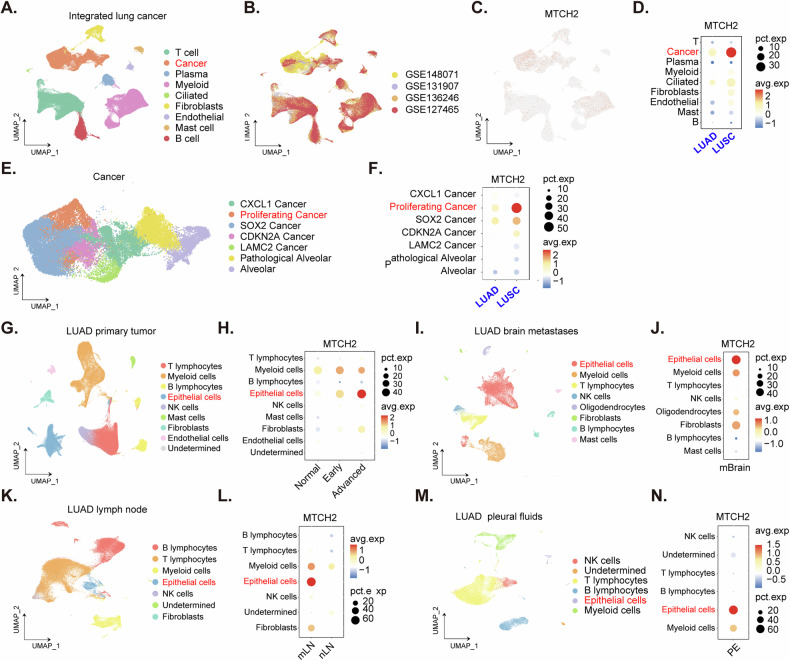
Single cell sequence data shows MTCH2 overexpression in cancer cell pollution of NSCLC mass. The UMAP (Uniform Manifold Approximation and Projection) plot of cell annotations derived from single-cell data of NSCLC illustrates the distribution of *MTCH2* expression (**A** and **B**). This distribution is further visualized in a Dot plot (**C**) and an expression density diagram (**D**) for LUAD or LUSC samples. The cancer cell population of the NSCLC tissues was isolated and subsequently grouped (**E**), with the expression distribution of *MTCH2* shown (**F**). The UMAP plots and expression density diagrams of cell annotations from original LUAD tissues (**G** and **H**), brain-metastatic LUAD tissues (**I** and **J**), lymph node-metastatic LUAD tissues (**K** and **L**), and LUAD pleural fluid (**M** and **N**) also illustrate the expression distribution of *MTCH2*.

The available single-cell data was utilized for further comprehensive analysis of *MTCH2* expression in NSCLC. Cell annotations, as provided by the original authors [41], were employed to ensure accuracy and consistency in the identification of cell types. A dimensionality reduction diagram illustrates these cell annotations, distinguishing between cancer cells and various non-cancerous cells in NSCLC mass (Fig. 2A). Additionally, a separate dimensionality reduction diagram depicts the source of the integrated data (Fig. 2B).These analysis revealed significant findings regarding *MTCH2* expression. A dot plot (Fig. 2C) and an expression density diagram (Fig. 2D) both indicate that *MTCH2* is highly expressed in cancer cell populations, with significantly higher levels in both LUAD and LUSC cells (Fig. 2C, D).The cancer cell population was further isolated and subsequently grouped (Fig. 2E). This analysis demonstrated that *MTCH2* expression is particularly elevated in the proliferative cancer cell population (Fig. 2E, F). These findings underscore the importance of *MTCH2* in the context of cancer cell proliferation and highlight its potential as a target for therapeutic intervention.

NSCLC single-cell data from GSE131907 was also employed for further in-depth analysis. The cell annotations, as provided by the original authors [42], were utilized to ensure precise cell type identification. Analysis revealed that *MTCH2* mRNA is highly expressed in epithelial cell populations of cancer tissues (Fig. 2G, H). *MTCH2* expression in epithelial cells progressively increases from normal tissues to early-stage cancer tissues, and further to advanced cancer tissues (Fig. 2G, H). Additionally, single-cell sequencing data from brain-metastatic NSCLC (Fig. 2I) indicated that *MTCH2* is overexpressed in epithelial cell populations of metastatic LUAD tissues (Fig. 2I, J). Similarly, single-cell sequencing data from lymph node-metastatic LUAD tissues (Fig. 2K) confirmed overexpression of *MTCH2* in epithelial cell populations (Fig. 2K, L). Furthermore, analysis of LUAD pleural fluid data also demonstrated that *MTCH2* is highly expressed in epithelial cell populations (Fig. 2M, N). These comprehensive findings consistently support the specific overexpression of MTCH2 in cancer cells within NSCLC mass, emphasizing its potential role as a critical marker and therapeutic target in the progression of NSCLC.

### Elevated MTCH2 expression in NSCLC tissues of locally-treated patients and various NSCLC cells

**Fig. 3:**
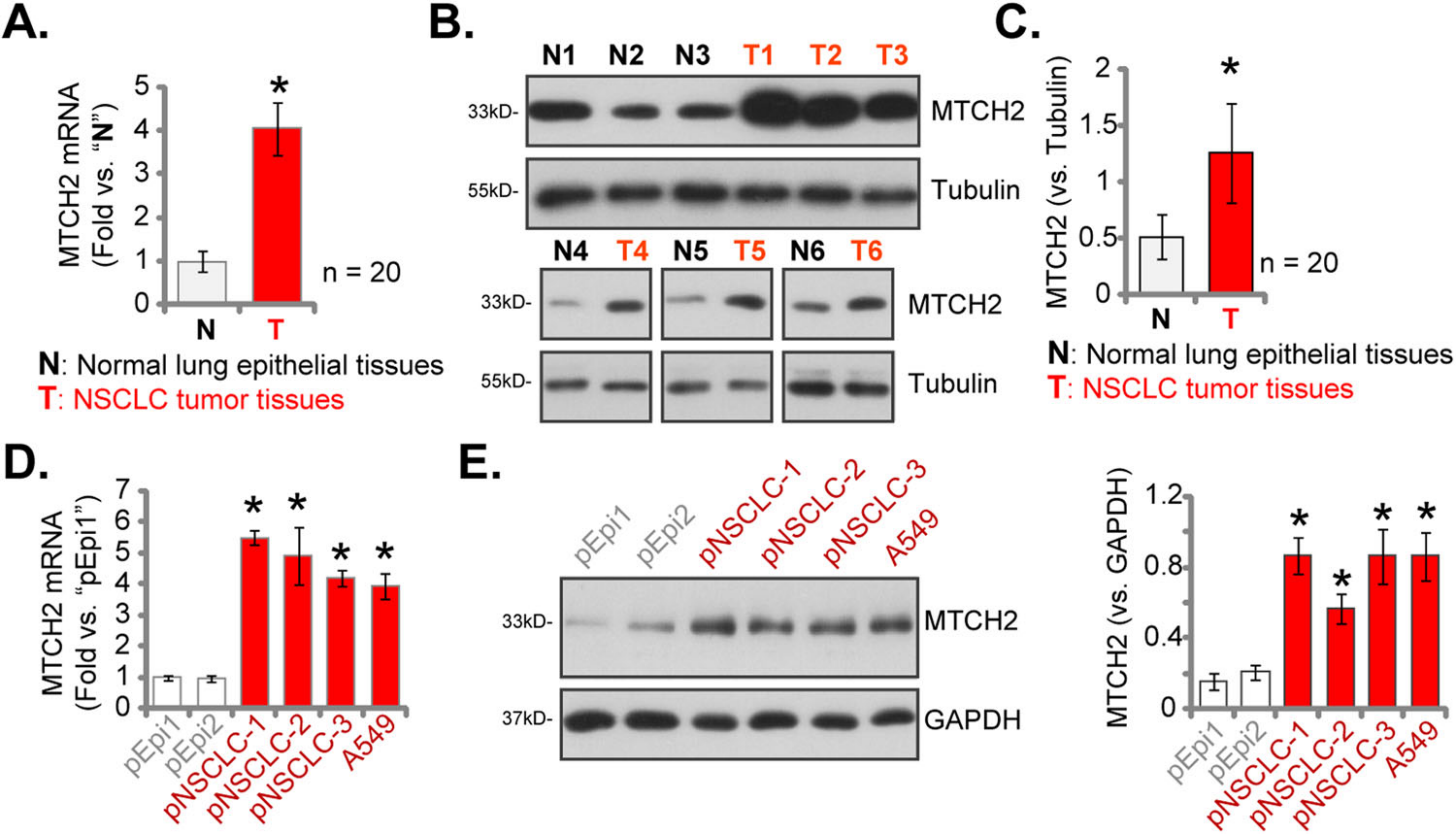
Elevated MTCH2 expression in NSCLC tissues of locally-treated patients and various NSCLC cells. The expression levels of *MTCH2* mRNA and protein were analyzed in NSCLC tumor tissues (denoted as “T”) and their corresponding adjacent normal lung epithelial tissues (denoted as “N”) collected from twenty primary NSCLC patients (**A**–**C**). Additionally, the levels of MTCH2 mRNA and protein were assessed in the described NSCLC cells and primary lung epithelial cells (**D** and **E**). The numerical values are presented as the mean ± standard deviation (SD) Statistical significance is indicated by *P < 0.05 compared to “N” tissues (Student’s *t* test) or “pEpi1” cells (ANOVA with Dunnett’s test).

To further investigate the expression profile of MTCH2 in NSCLC tissues of locally-treated patients, we performed a comprehensive evaluation. We obtained NSCLC tumor specimens (designated as “T”) and their corresponding adjacent non-cancerous lung epithelial tissues (designated as “N”) from a cohort of twenty primary NSCLC patients (LUAD, stages III-IV). Through careful examination of the tissue lysates, we confirmed a significant elevation in *MTCH2* mRNA expression levels in the NSCLC tissues relative to the adjacent normal tissues (Fig. 3A). Moreover, the analysis of MTCH2 protein expression revealed an upregulation in tumor tissues from six representative patients (labeled as “T1” to “T6”) (Fig. 3B). Aggregating the data from all twenty patient samples, our analysis demonstrated a statistically significant upregulation of MTCH2 protein expression in the NSCLC tissues compared to the normal lung epithelial counterparts (Fig. 3C).

Subsequent experimental analyses were performed to explore the expression levels of *MTCH2* across various NSCLC cells. It included primary human NSCLC cells, specifically “pNSCLC-1/-2/-3,” derived from three patients as previously documented [26, 36], alongside the immortalized A549 cell line. The results demonstrated a marked elevation in *MTCH2* mRNA expression within both primary and immortalized NSCLC cells, when compared to primary human lung epithelial cells, designated “pEpi1” and “pEpi2” from two separate donors [24] (Fig. 3D). In addition, upregulation of MTCH2 protein was detected across all these primary and immortalized NSCLC cells (Fig. 3E), which stood in contrast to the significantly lower expression observed in the lung epithelial cells (Fig. 3E). These findings further corroborate the heightened expression of MTCH2 in NSCLC tissues and cells, suggesting a potential role for MTCH2 in the pathogenesis and progression of NSCLC.

### MTCH2 silencing inhibits NSCLC cell viability, proliferation and migration

**Fig. 4:**
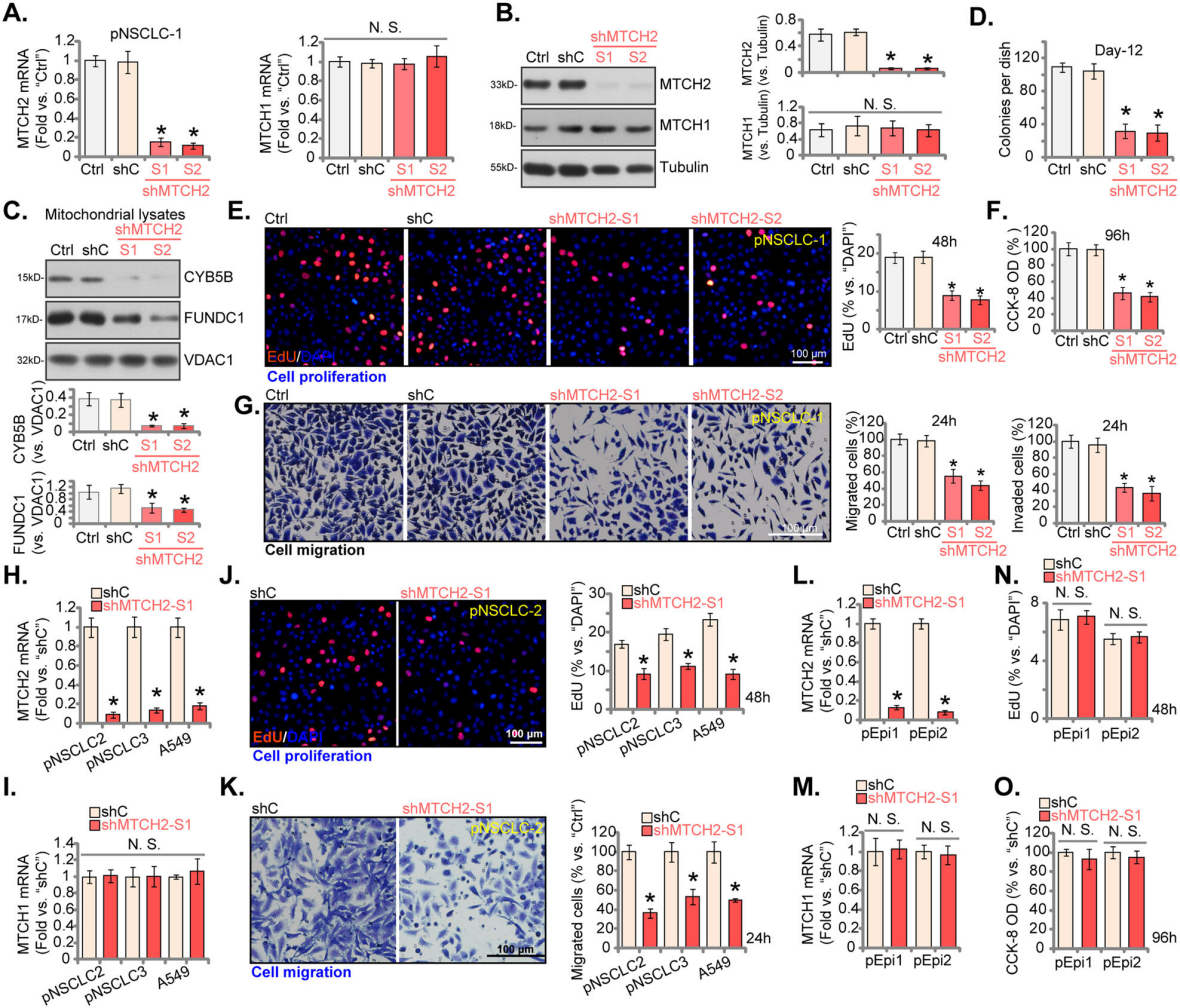
MTCH2 silencing inhibits NSCLC cell viability, proliferation and migration. The primary pNSCLC-1 cells were individually treated with specific MTCH2 shRNAs (shMTCH2-S1 and shMTCH2-S2, representing distinct sequences) or a control scramble non-sense shRNA (shC). The expression levels of MTCH1 and MTCH2 (both mRNA and protein) were assessed (**A** and **B**). Expression of listed proteins in the mitochondrial fraction lysates was also shown (**C**). Equal numbers of these cells were cultured for specific durations to evaluate various cellular function, including colony formation (**D**), cell proliferation (percentage of EdU-incorporated nuclei, **E**), viability (CCK-8 OD, **F**), in vitro cell migration (Transwell assays) and invasion (Matrigel Transwell assays) (**G**). Additionally, stable cells derived from other primary NSCLC cells (pNSCLC-2 and pNSCLC-3), the A549 immortalized cells, or the lung epithelial cells (pEpi1 or pEpi2), expressing either shC or shMTCH2-S1, were established and examined for *MTCH2* and *MTCH1* mRNA expression (**H**, **I**, **L**, and **M**). Equal numbers of these cells were cultured for specific durations to assess cell proliferation (**J** and **N**), migration (**K**), and viability (**O**) using the same methods. The numerical values are presented as the mean ± standard deviation (SD, *n* = 5). “Ctrl” represents parental control cells. Statistical significance is indicated by *P < 0.05 compared to “shC” cells (ANOVA with Dunnett’s test), while “N.S.” denotes non-statistically significant differences (*P* > 0.05) (ANOVA with Dunnett’s test). The experiments shown in this figure were repeated five times (all biological repeats), consistently yielding similar results. Scale bar = 100 μm.

To elucidate the functional role of MTCH2 in NSCLC cells, we employed the shRNA-mediated knockdown approach. Lentiviral vectors carrying MTCH2-specific shRNAs were transduced into pNSCLC-1 primary NSCLC cells (as previously described [26, 36, 37]), followed by puromycin selection to generate stable cells. Two distinct shRNAs targeting MTCH2, designated “shMTCH2-S1” and “shMTCH2-S2”, with non-overlapping sequences, were utilized, resulting in significant reductions in both *MTCH2* mRNA (Fig. 4A) and protein (Fig. 4B) levels. MTCH1 expression remained unaffected (Fig. 4A, B). Levels of CYB5B (cytochrome b5 type B) and FUNDC1 (FUN14 domain containing 1), two MTCH2-dependent mitochondrial proteins [30], were significantly reduced in the mitochondria of MTCH2-silenced pNSCLC-1 cells (Fig. 4C). The depletion of MTCH2 via these shRNAs impeded pNSCLC-1 cell proliferation, inhibiting colony formation (Fig. 4D), and decreasing the proportion of EdU-positive nuclei (Fig. 4E). Additionally, cell viability, measured by CCK-8 assay, was markedly decreased in MTCH2-silenced pNSCLC-1 cells (Fig. 4F). Moreover, MTCH2 knockdown significantly impaired the motility of pNSCLC-1 cells, with significant reductions in migration and invasion capacities (Fig. 4G). Importantly, a scrambled control shRNA (“shC”) did not result in significant changes in MTCH1/2 expression (Fig. 4A, B) or the functional properties of pNSCLC-1 cells (Fig. 4C–G).

To assess the reproducibility of MTCH2 silencing across different primary NSCLC cells, lentiviral vectors expressing shMTCH2-S1 were introduced into primary human NSCLC cells from other two patients, specifically pNSCLC-2, pNSCLC-3, and also into the A549 immortalized cell line. Stable cells were selected using puromycin. The results demonstrated a significant reduction in *MTCH2* mRNA expression in these NSCLC cells treated with shMTCH2-S1 (Fig. 4H), with *MTCH1* mRNA levels remaining unaffected (Fig. 4I). Consistent with observations in pNSCLC-1 cells, MTCH2 knockdown via shMTCH2-S1 significantly inhibited cell proliferation (as indicated by decreased EdU incorporation, Fig. 4J) and reduced cell migration (Fig. 4K) in both primary and immortalized NSCLC cells. The application of shMTCH2-S1 in primary human lung epithelial cells, pEpi1 and pEpi2, effectively suppressed *MTCH2* mRNA expression (Fig. 4L), without affecting *MTCH1* mRNA expression (Fig. 4M). Despite this, no significant effect on cell proliferation (nuclear EdU incorporation) was observed in the lung epithelial cells (Fig. 4N). Furthermore, the epithelial cell viability, tested via the CCK-8 assay, showed no significant inhibition following MTCH2 knockdown with shMTCH2-S1 (Fig. 4O). Together these results showed that MTCH2 silencing inhibited NSCLC cell viability, proliferation and migration.

### MTCH2 silencing induces apoptosis activation in NSCLC cells

**Fig. 5:**
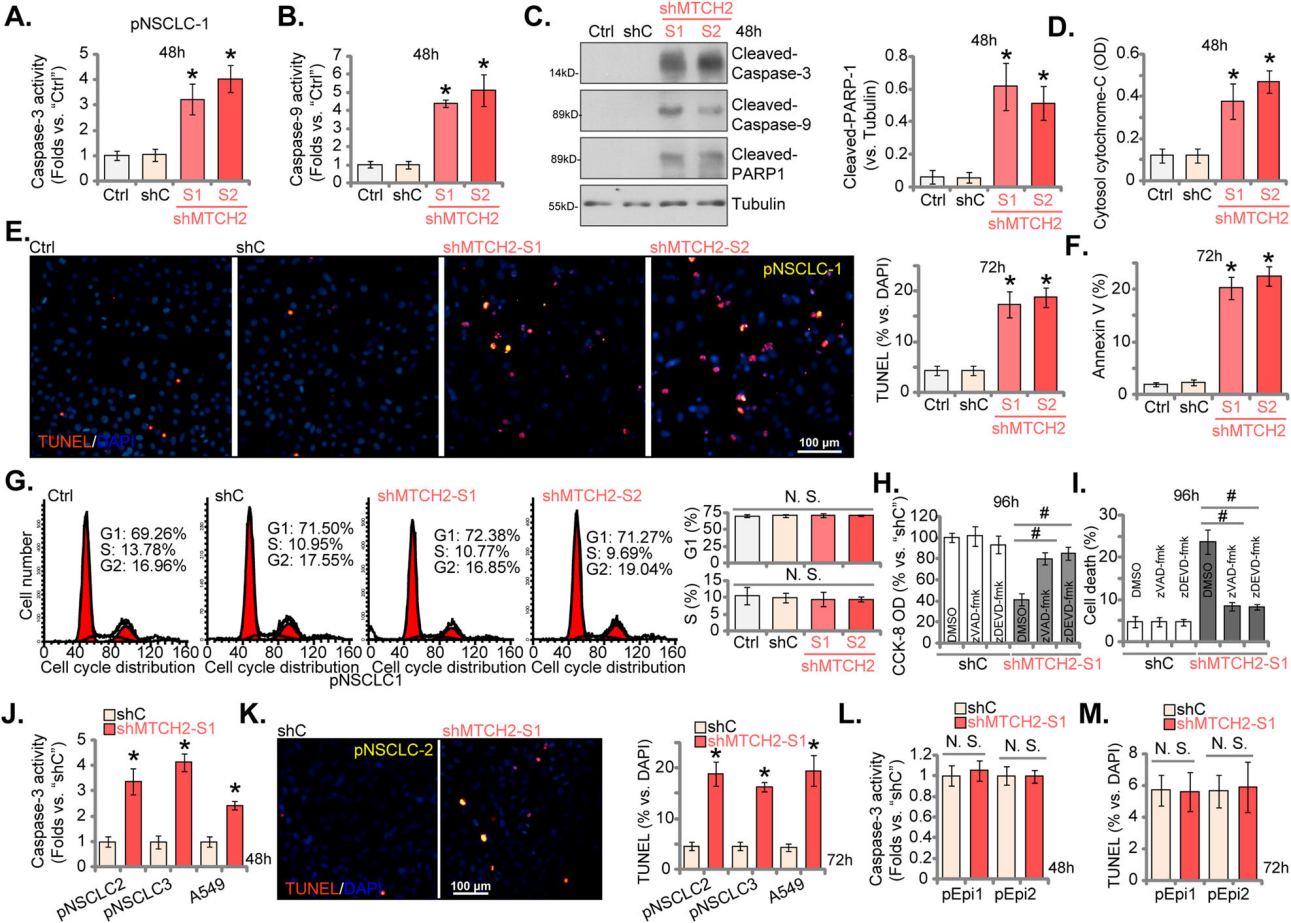
MTCH2 silencing induces apoptosis activation in NSCLC cells. The primary pNSCLC-1 cells were individually treated with specific MTCH2 shRNAs (shMTCH2-S1 and shMTCH2-S2, representing distinct sequences) or a control scramble non-sense shRNA (shC). Equal numbers of these cells were cultured for specific durations, the Caspase-3 activity (**A**), the Caspase-9 activity (**B**), expression of apoptosis-related proteins (**C**) and cytosol cytochrome C contents (**D**) were tested; Cell apoptosis was measured via measuring the percentage of nuclei incorporating TUNEL staining (**E**) or through Annexin V-PI FACS assays (**F**). Cell cycle distribution was also analyzed (**G**). The primary pNSCLC-1 cells with shMTCH2-S1 or shC were co-treated with zDEVD-fmk (50 μM), zVAD-fmk (50 μM)or the vehicle control (0.15% of DMSO) for 96 h, cell viability and death were tested via CCK-8 (**H**) and medium LDH releasing (**I**) assays, respectively. Additionally, stable cells derived from other primary NSCLC cells (pNSCLC-2 and pNSCLC-3), the A549 immortalized cells, or the lung epithelial cells (pEpi1 or pEpi2), expressing either shC or shMTCH2-S1, were established and were cultured for specific durations, the Caspase-3 activity (**J** and **L**) and apoptosis (nuclear TUNEL staining, **K** and **M**) were measured. The numerical values are presented as the mean ± standard deviation (SD, *n* = 5). “Ctrl” represents parental control cells. Statistical significance is indicated by *P < 0.05 compared to “shC” cells, while “N.S.” denotes non-statistically significant differences (*P* > 0.05) (ANOVA with Dunnett’s test). ^#^*P* < 0.05 (**H** and **I**) (ANOVA with Dunnett’s test). The experiments shown in this figure were repeated five times (all biological repeats), consistently yielding similar results. Scale bar = 100 μm.

The shRNA-mediated knockdown of MTCH2 demonstrated significant inhibitory effects on cell viability, proliferation, and migration in both primary and immortalized NSCLC cells. To further elucidate the functional implications of MTCH2, we assessed its impact on cell apoptosis. pNSCLC-1 cells subjected to shMTCH2-S1 or shMTCH2-S2 treatment showed a marked elevation in Caspase-3 activity (Fig. 5A) and Caspase-9 activity (Fig. 5B). MTCH2 silencing also resulted in the cleavage of both Caspase-3 and poly (ADP-ribose) polymerase-1 (PARP1) in these cells (Fig. 5C). Furthermore, cytosolic levels of cytochrome-C, a key marker of apoptotic activation, were increased in MTCH2-shRNA-expressing pNSCLC-1 cells (Fig. 5D). This cascade of events triggered by MTCH2 knockdown culminated in heightened apoptosis in pNSCLC-1 cells, as indicated by a significant rise in nuclei exhibiting positive TUNEL staining (Fig. 5E) and a higher percentage of Annexin V-positive pNSCLC-1 cells (Fig. 5F). In contrast, the control treatment with shC did not induce apoptosis in pNSCLC-1 cells (Fig. 5A–F). Notably, shRNA-induced silencing of MTCH2 did not significantly alter cell cycle progression in primary NSCLC cells, as shown in Fig. 5G.

Additionally, the use of two established apoptosis inhibitors, including the pan-caspase inhibitor z-VAD-fmk and the Caspase-3 inhibitor z-DEVD-fmk, significantly mitigated the shMTCH2-S1-induced reduction in cell viability (Fig. 5H) and cell death (Fig. 5I) in pNSCLC-1 cells. Cell death was tested via quantifying medium LDH levels (Fig. 5I). In additional primary human NSCLC cells (pNSCLC-2 and pNSCLC-3), as well as in the immortalized A549 cells, the creation of stable MTCH2 knockdown through the use of the shMTCH2-S1-expressing lentiviral particles (see Fig. 4) similarly induced Caspase-3 activation (Fig. 5J) and elevated the number of nuclei exhibiting positive TUNEL staining (Fig. 5K), thus affirming the induction of apoptosis. However, in primary human lung epithelial cells, “pEpi1” and “pEpi2,” MTCH2 knockdown via shMTCH2-S1 (see Fig. 4) did not result in increased Caspase-3 activity (Fig. 5L) and n increase in TUNEL-positive nuclei ratio (Fig. 5M). This indicates a cancer cell-type-specific response to MTCH2 silencing in apoptosis induction.

### MTCH2 silencing impairs mitochondrial function in NSCLC cells

**Fig. 6:**
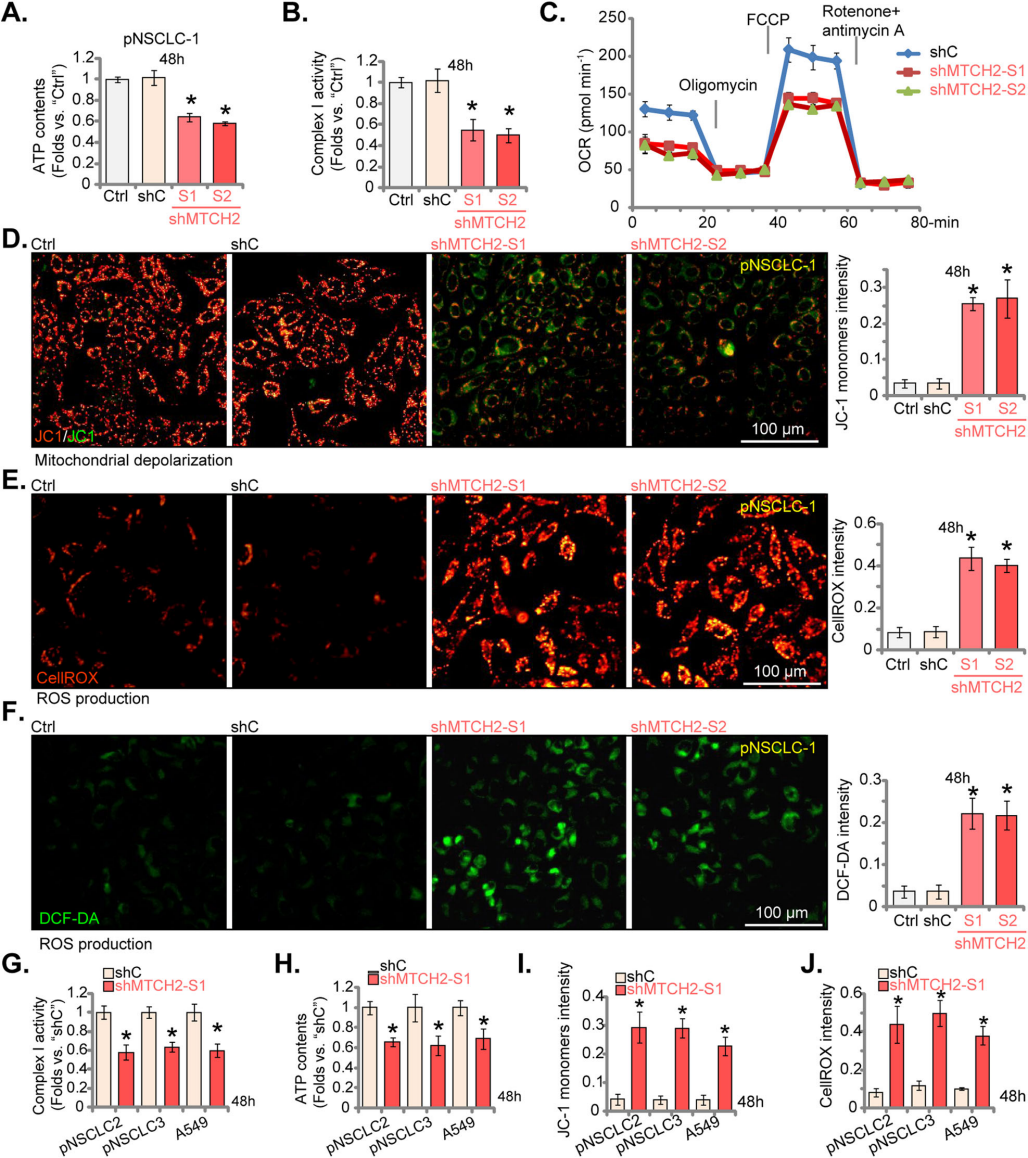
MTCH2 silencing impairs mitochondrial function in NSCLC cells. The primary pNSCLC-1 cells were individually treated with specific MTCH2 shRNAs (shMTCH2-S1 and shMTCH2-S2, representing distinct sequences) or a control scramble non-sense shRNA (shC). Equal numbers of these cells were cultured for specific durations, and the following parameters were tested, including the mitochondrial complex-I activity (**A**), ATP contents (**B**), oxygen consumption rate (OCR, tested via the Seahorse assays, **C**), mitochondrial depolarization (tested via JC-1 green monomer intensity, **D**), and ROS levels (measured using CellROX and DCF-DA fluorescence intensity, **E** and **F**). Stable cells derived from other primary NSCLC cells (pNSCLC-2 and pNSCLC-3), the A549 immortalized cells expressing either shC or shMTCH2-S1 were established and were cultured for specific durations, the mitochondrial complex-I activity (**G**), ATP contents (**H**), mitochondrial depolarization (**I**) and ROS levels (**J**) were tested similarly. The numerical values are presented as the mean ± standard deviation (SD, *n* = 5). “Ctrl” represents parental control cells. Statistical significance is indicated by *P < 0.05 compared to “shC” cells (ANOVA with Dunnett’s test). The experiments shown in this figure were repeated five times (all biological repeats), consistently yielding similar results. Scale bar = 100 μm.

We next tested whether MTCH2 silencing impaired mitochondrial function in NSCLC cells. To support the mitochondrial dysfunction, we identified a significant reduction in mitochondrial complex-I activity in MTCH2-silenced pNSCLC-1 cells (Fig. 6A), accompanied by decreased ATP levels (Fig. 6B). The results from the Seahorse assay indicated that MTCH2 shRNA substantially suppressed both basal and maximal oxygen consumption rates (OCR) in pNSCLC-1 cells (Fig. 6C). Furthermore, MTCH2 knockdown triggered mitochondrial depolarization, as evidenced by the shift of JC-1 fluorescence from red aggregates to green monomers (Fig. 6D). In MTCH2-silenced pNSCLC-1 cells, there was a significant increase in reactive oxygen species (ROS) production, demonstrated by the elevated CellROX red fluorescence intensity (Fig. 6E) and DCF-DA green fluorescence intensity (Fig. 6F). Conversely, treatment with shC did not impair mitochondrial function in pNSCLC-1 cells (Fig. 6A–F).These findings underscore the extensive mitochondrial damage resulting from MTCH2 silencing in primary pNSCLC-1 cells.

In additional primary human NSCLC cells (pNSCLC-2 and pNSCLC-3), as well as in the immortalized A549 cell line, the establishment of stable MTCH2 knockdown using the shMTCH2-S1-expressing lentiviral vector (see Figs. 4 and 5) similarly resulted in a significant decrease in mitochondrial complex-I activity (Fig. 6G) and cellular ATP levels (Fig. 6H). The transition to JC-1 green fluorescent monomers further corroborated the occurrence of mitochondrial depolarization in these cells (Fig. 6I). shMTCH2-S1 also resulted induced ROS production, evidenced by the increased CellROX intensity in the NSCLC cells (Fig. 6J). These consistent findings across multiple NSCLC cell types support the critical role of MTCH2 in maintaining mitochondrial function.

### MTCH2 knockout (KO) impairs mitochondrial function and inhibits NSCLC cell progression

**Fig. 7:**
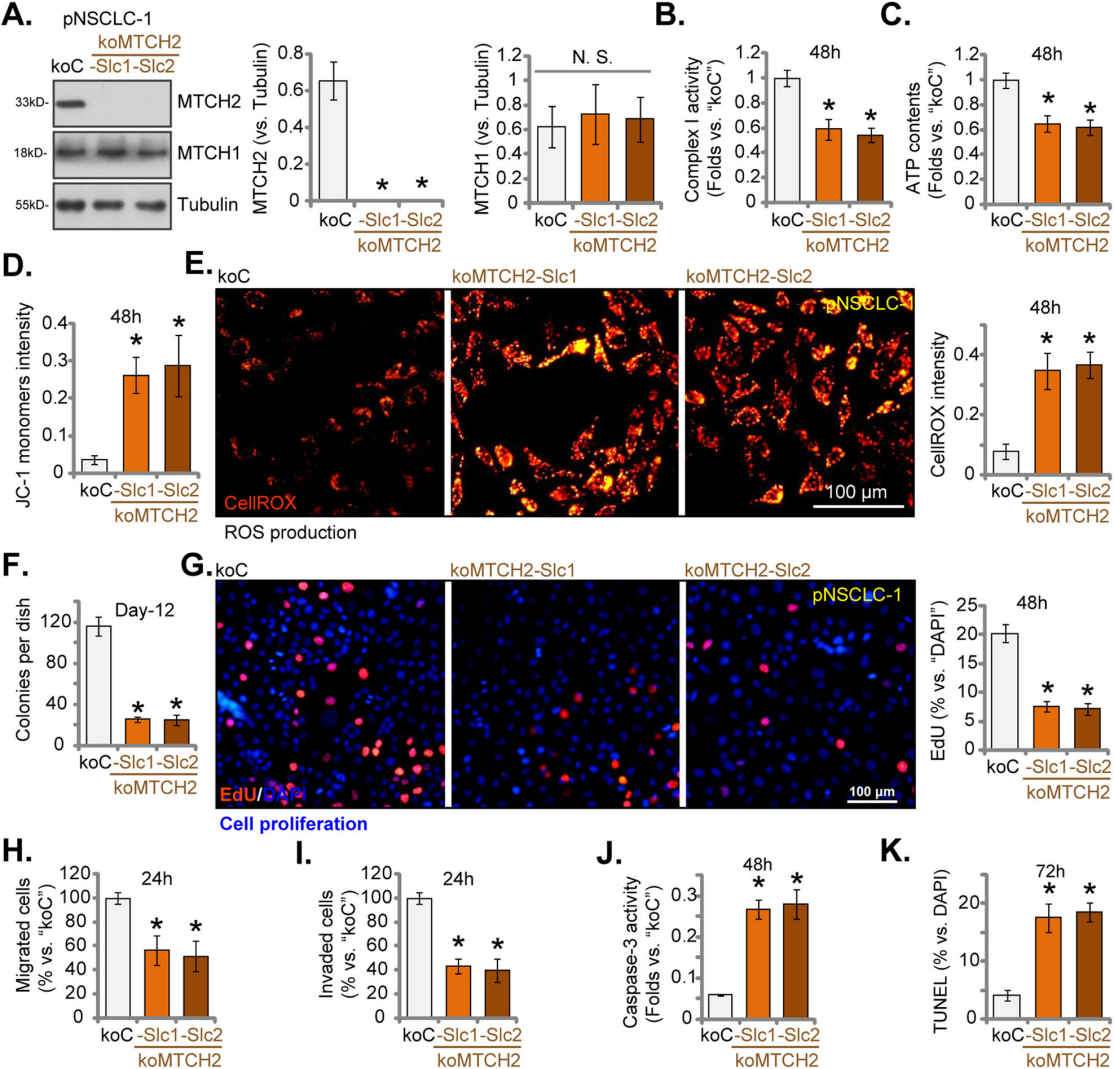
MTCH2 knockout (KO) impairs mitochondrial function and inhibits NSCLC cell progression. The “koMTCH2” pNSCLC-1 cells, including two sub-clones, koMTCH2-Slc1 and koMTCH2-Slc2, were created by introducing the CRISPR-MTCH2-KO construct into Cas9-expressing pNSCLC-1 cells. Control cells, referred to as “koC,” were established by treating the Cas9-expressing pNSCLC-1 cells with the CRISPR-KO control construct. MTCH2 and MTCH1 protein expression was shown (**A**). Equal numbers of these cells were cultured for specific durations to evaluate mitochondrial function, including the mitochondrial complex-I activity (**B**), ATP content (**C**), mitochondrial depolarization (teste via JC-1 green monomer intensity, **D**) and ROS levels (measured using CellROX fluorescence intensity, **E**); Various cellular processes, including colony formation (**F**), cell proliferation (percentage of EdU-incorporated nuclei, **G**), in vitro cell migration (Transwell assays, **H**), and invasion (Matrigel Transwell assays, **I**) were also tested. Cell apoptosis was measured via the Caspase-3 activity assay (**J**) and nuclear TUNEL staining (**K**) assays. The numerical values are presented as the mean ± standard deviation (SD, *n* = 5). Statistical significance is indicated by *P < 0.05 compared to “koC” cells, while “N.S.” denotes non-statistically significant differences (*P* > 0.05) (ANOVA with Dunnett’s test). The experiments shown in this figure were repeated five times (all biological repeats), consistently yielding similar results. Scale bar = 100 μm.

To mitigate potential off-target effects induced by the applied MTCH2 shRNAs and ensure a thorough KO of MTCH2, the CRISPR/Cas9 approach was employed. Specifically, the CRISPR/Cas9-MTCH2-KO puro-construct-expressing lentiviral particles were added into Cas9-expressing pNSCLC-1 cells, as detailed in a prior study [36]. After puromycin selection, single stable cells were isolated and subjected to MTCH2 KO screening, which led to the establishment of single-cell-derived NSCLC-1 MTCH2 KO selections: “koMTCH2-Slc1” and “koMTCH2-Slc2”. Western blotting assays validated the complete elimination of MTCH2 protein in koMTCH2 pNSCLC-1 cells (Fig. 7A). In contrast, MTCH1 protein expression remained unaffected (Fig. 7A).

In line with the observations from MTCH2-silenced cells, mitochondrial complex-I activity (Fig. 7B) and ATP levels (Fig. 7C) were significantly reduced in MTCH2 KO pNSCLC-1 cells. Furthermore, MTCH2 KO led to mitochondrial depolarization, as indicated by the enhance of JC-1 monomer intensity (Fig. 7D). The increased CellROX intensity suggested elevated ROS production in MTCH2 KO NSCLC cells (Fig. 7E). Further assays demonstrated that MTCH2 KO exerted substantial inhibitory effects on pNSCLC-1 cell proliferation, as shown by reduced colony formation (Fig. 7F) and decreased EdU-positive nuclei staining (Fig. 7G). Additionally, in vitro cell migration (Fig. 7H) and invasion (Fig. 7I) assays revealed that the koMTCH2-Slc1 and koMTCH2-Slc2 pNSCLC-1 cells exhibited significantly diminished capabilities. MTCH2 KO also induced apoptosis in the pNSCLC-1 cells, evidenced by increased caspase-3 activity (Fig. 7J) and a higher nuclear TUNEL ratio (Fig. 7K). These results reinforce the conclusion that MTCH2 plays a critical role in maintaining mitochondrial integrity and cellular processes in pNSCLC-1 cells.

### MTCH2 overexpression augments NSCLC cell proliferation and migration

**Fig. 8:**
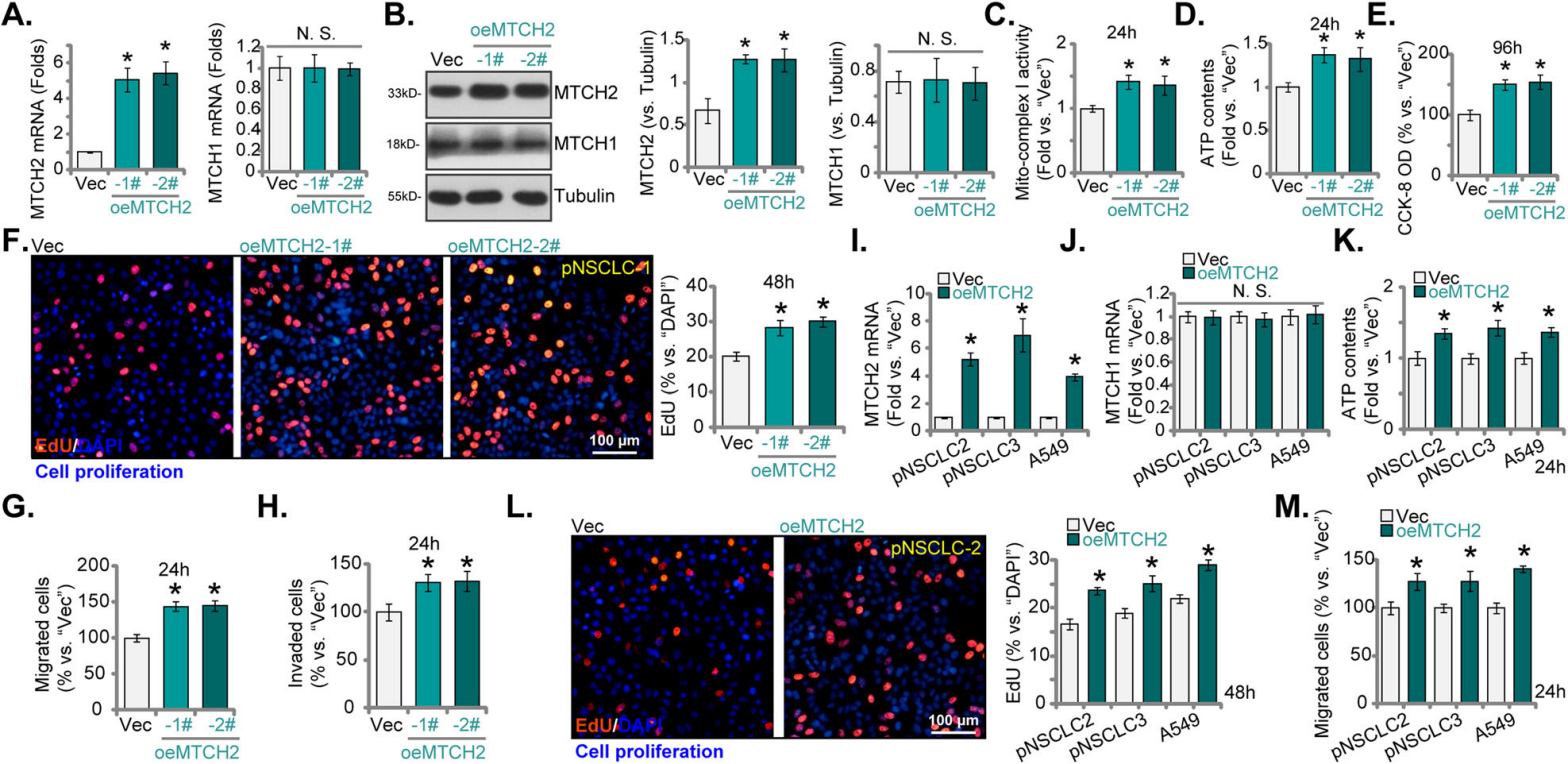
MTCH2 overexpression augments NSCLC cell proliferation and migration. pNSCLC-1 cells expressing the MTCH2-overexpressing construct, specifically “oeMTCH2-1#“ and “oeMTCH2-2#“ (representing two stable cell selections), along with those containing the empty vector (“Vec”), were generated. MTCH2 and MTCH1 expression levels were tested at both mRNA and protein levels (**A** and **B**). Equal numbers of these cells were cultured for specific durations to evaluate various cellular functions, including mitochondrial complex I activity (**C**), cellular ATP content (**D**), cell viability (CCK-8 OD, **E**), cell proliferation (measured by the percentage of EdU-incorporated nuclei, **F**), in vitro cell migration (“Transwell” assays, **G**) and invasion (“Matrigel Transwell”, **H**). Additionally, stable cells derived from other primary NSCLC cells (pNSCLC-2 and pNSCLC-3) or A549 immortalized cells, expressing either the MTCH2 construct (“oeMTCH2”) or the empty vector (“Vec”), were established. These cells were examined for *MTCH2* and *MTCH1* mRNA expression (**I** and **J**). Equal numbers of these cells were cultured for specific durations to assess cellular ATP content (**K**), cell proliferation (**L**), and migration (**M**) using the same methods. The numerical values are presented as the mean ± standard deviation (SD, *n* = 5). Statistical significance is indicated by *P < 0.05 compared to “Vec” cells, while “N.S.” denotes non-statistically significant differences (*P* > 0.05) (ANOVA with Dunnett’s test). The experiments shown in this figure were repeated five times (all biological repeats), consistently yielding similar results. Scale bar = 100 μm.

The results described above unequivocally demonstrate the significant anti-cancer effects resulting from the knockdown or KO of MTCH2 in both primary and immortalized NSCLC cells. This observation prompted us to hypothesize that ectopic overexpression of MTCH2 might produce opposite outcomes. To test the hypothesis, we introduced a lentiviral construct containing the MTCH2-expressing sequence (“oeMTCH2”) into pNSCLC-1 cells. Following puromycin selection, we established two stable cell selections, designated as “oeMTCH2-1#” and “oeMTCH2-2#.” In comparison to control pNSCLC-1 cells harboring an empty vector (“Vec”), we observed a substantial increase in both *MTCH2* mRNA (Fig. 8A) and protein (Fig. 8B) levels in the oeMTCH2 cells. The expression of MTCH1 remained unchanged (Fig. 8A, B). As illustrated, the ectopic overexpression of MTCH2 significantly enhanced the mitochondrial function of pNSCLC-1 cells, as evidenced by the increased activity of mitochondrial complex I (Fig. 8C) and elevated cellular ATP levels (Fig. 8D). The proliferation of pNSCLC-1 cells was enhanced upon MTCH2 overexpression, demonstrated by the increase in CCK-8 OD values (Fig. 8E) and a higher percentage of EdU-positive nuclei (Fig. 8F). Additionally, MTCH2 overexpression increased the number of migrated (Fig. 8G) and invaded pNSCLC-1 cells (Fig. 8H).

Subsequently, the lentiviral vector encoding the MTCH2-expressing construct (“oeMTCH2”) was transduced into additional primary NSCLC cells (pNSCLC-2 and pNSCLC-3) as well as the immortalized A549 cell line, resulting in the establishment of stable cells following puromycin selection. In these modified NSCLC cells, substantial elevation in *MTCH2* mRNA levels was observed (Fig. 8I), with *MTCH1* mRNA levels remained unaffected (Fig. 8J). The overexpression of MTCH2 significantly increased the cellular ATP contents in these NSCLC cells (Fig. 8K). Moreover, MTCH2 overexpression enhanced cell proliferation (Fig. 8L) and promoted marked acceleration of in vitro cell migration (Fig. 8M) in both primary and immortalized NSCLC cells. These results again underscore the role of MTCH2 in augmenting mitochondrial function, and the proliferative and migratory capacities of NSCLC cells.

### MTCH2 silencing hinders the growth of subcutaneous NSCLC xenografts in nude mice

To explore the potential impact of MTCH2 on the growth of NSCLC cells in vivo, primary pNSCLC-1 cells were administered subcutaneously into the flanks of nude mice, with each mouse receiving six million cells. Over a span of three weeks, pNSCLC-1 xenografts formed, achieving a size of approximately 100 mm³ by “Day-0”. The mice with these xenografts were then randomly assigned to two groups. The treatment group was given intratumoral injections of adeno-associated virus (aav) carrying shMTCH2-S1 (“aav-shMTCH2-S1”), while the control group received aav with scramble control shRNA (“aav-shC”). These virus injections were repeated after 72 h and tumor sizes were monitored every five days.

**Fig. 9:**
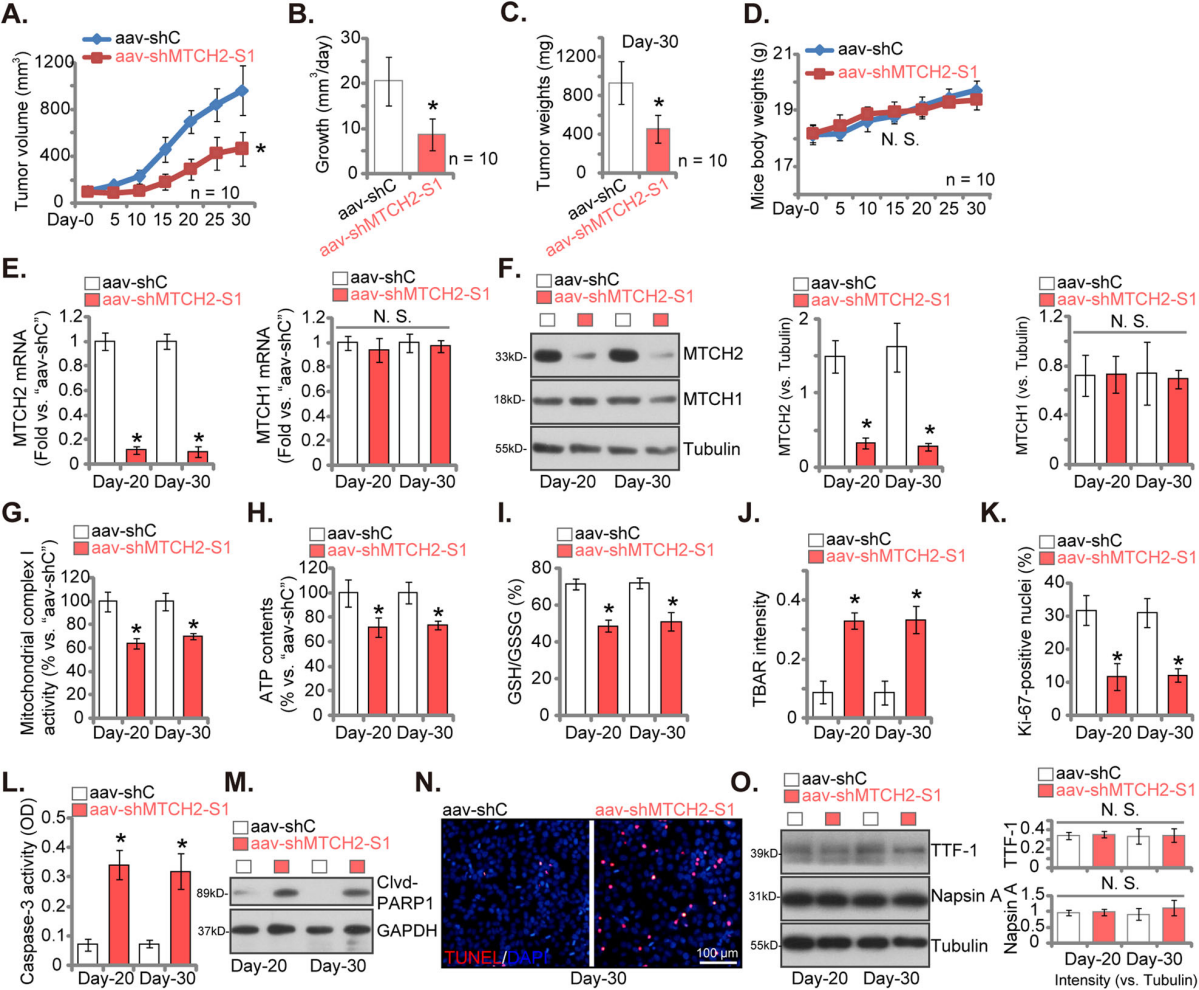
MTCH2 silencing hinders the growth of subcutaneous NSCLC xenografts in nude mice. The nude mice implanted with pNSCLC-1 xenografts received intratumoral injections of either adeno-associated virus encoding shMTCH2-S1 (“aav-shMTCH2-S1”) or a control shRNA adeno-associated virus (“aav-shC”). The volumes of the pNSCLC-1 xenografts (**A**) and the body weights of the mice (**D**) were recorded every five days. The daily growth rate of the pNSCLC-1 xenografts was determined (**B**). On Day-30, all pNSCLC-1 xenografts were harvested and weighed (**C**). Tissue lysates from the described xenografts were examined for specific mRNA and protein expressions (**E**, **F**, **M** and **O**). Additionally, assessments included mitochondrial complex-I activity (**G**), ATP levels (**H**), the GSH/GSSG ratio (**I**), TBAR intensity (**J**), and Caspase-3 activity (**L**). The pNSCLC-1 tumor sections also underwent IHC staining for Ki-67, with the Ki-67-positive nuclei ratio quantified (**K**). Furthermore, the sections were subjected to the fluorescence staining for TUNEL/DAPI, with representative images presented (**N**). Data are presented as mean ± standard deviation (SD). Statistical significance was denoted by *P < 0.05 compared to the “aav-shC” treatment, while “N. S.” indicates non-significant differences (*P* > 0.05). For panels **A**–**D**, each group consisted of ten mice (*n* = 10) (ANOVA with Dunnett’s test for **A**, and Student’s *t* test for **B**–**D**). In panels **E**–**N**, five random tissue samples from each xenograft were analyzed (*n* = 5) (ANOVA with Dunnett’s test). Scale bar = 100 μm.

The administration of aav-shMTCH2-S1 significantly impeded the growth of pNSCLC-1 xenografts in nude mice, resulting in markedly smaller tumor volumes (Fig. 9A). Utilizing the methods described early [26, 36], the daily growth rate of pNSCLC-1 xenografts was calculated in mm³ per day (Fig. 9B). The data clearly demonstrated a remarkable reduction in the growth of pNSCLC-1 xenografts after aav-shMTCH2-S1 treatment (Fig. 9B). By the conclusion of the animal study on Day-30, all pNSCLC-1 xenografts were excised and individually weighed. The pNSCLC-1 xenografts from the aav-shMTCH2-S1 group were both lighter and smaller compared to those from the aav-shC group (Fig. 9C). In contrast, there were no significant differences in the body weights of the mice among two groups (Fig. 9D). These results provided compelling evidence for the inhibitory effect of aav-shMTCH2-S1 on the growth of NSCLC xenografts in nude mice.

On Day-20 and Day-30 following the initial virus injection, a single pNSCLC-1 xenograft was collected from each experimental group, totaling four xenografts. These specimens were dissected and homogenized, revealing significant reduction in both mRNA (Fig. 9E) and protein (Fig. 9F) levels of MTCH2 within the pNSCLC-1 xenograft tissues treated with aav-shMTCH2-S1. In contrast, the expression of MTCH1 remained unaffected (Fig. 9E, F). In MTCH2-silenced pNSCLC-1 xenograft tissues, both mitochondrial complex-I activity (Fig. 9G) and ATP contents (Fig. 9H) were significantly reduced. Additionally, the GSH/GSSH ratio decreased following aav-shMTCH2-S1 treatment (Fig. 9I), while TBAR activity, indicating lipid peroxidation levels, increased (Fig. 9J). These findings suggest that MTCH2 silencing led to mitochondrial dysfunction and oxidative injury in pNSCLC-1 xenograft tissues.

Significantly, the quantified IHC results there was a marked decrease in the ratio of nuclear Ki-67-positive staining observed in pNSCLC-1 xenografts treated with aav-shMTCH2-S1, indicating substantial suppression of proliferation in vivo (Fig. 9K). Further examination of the xenograft tissues revealed heightened Caspase-3 activity in those treated with aav-shMTCH2-S1 (Fig. 9L), accompanied by increased levels of cleaved-PARP1 (Fig. 9M). Additionally, immunofluorescence analysis of xenograft sections supported the induction of apoptosis in MTCH2-silenced pNSCLC-1 xenografts, as evidenced by a higher percentage of TUNEL-positive apoptotic nuclei (Fig. 9N). We also conducted tests for differentiation marker proteins and found no changes in the expression of thyroid transcription factor-1 (TTF-1) and Napsin A [43–45] in the MTCH2-silenced xenograft tissues. Together, these results support that aav-shMTCH2-S1 injection resulted in MTCH2 silencing, impairment of mitochondrial function, suppression of proliferation, and apoptosis in pNSCLC-1 xenografts.

## Discussion

Recent advancements in targeted therapies for NSCLC have significantly improved patient outcomes [9, 14, 17, 46]. Molecular profiling has led to the development of targeted treatments (inhibitors/antibodies) for specific genetic alterations, including EGFR, ALK, ROS1, BRAF, MET, and RET [9, 14, 17, 46]. The emergence of immune checkpoint inhibitors (e.g. PD-1/PD-L1 inhibitors) has further enhanced treatment options, particularly for patients without actionable mutations [47, 48]. Despite these significant advancements, targeted therapies for NSCLC face limitations such as the drug resistance and the variability of treatment response among patients with different genetic backgrounds [9, 14, 17, 46]. It is therefore important to explore further targets of NSCLC.

Mitochondrial function is closely linked to the growth and progression of NSCLC. Mitochondria are crucial for cellular energy production, and hyper-function can support the high metabolic demands of rapidly proliferating cancer cells [24–27]. Additionally, mitochondrial pathways are involved in apoptosis regulation. Recent studies have reported that several mitochondrial proteins are overexpressed in NSCLC, correlating with poor prognosis and other clinical parameters of the patients [24–26]. These mitochondrial proteins are essential for maintaining mitochondrial hyper-function, which supports NSCLC cell growth [24–26]. Xia et al., demonstrated that the overexpression of YME1L (YME1 Like 1 ATPase) in NSCLC is crucial for mitochondrial functionality and NSCLC cell growth [24]. Zhang et al., identified the mitochondrial protein aarF domain containing kinase 2 (ADCK2) as a potential therapeutic target for NSCLC [25]. Silencing or knockout of ADCK2 disrupted mitochondrial function and inhibited NSCLC cell growth [25]. Zhou et al., reported that the overexpression of POLRMT (RNA polymerase mitochondrial) is necessary for NSCLC cell growth, possibly by maintaining mitochondrial DNA content, mitochondrial transcripts, and mTOR activation [26].

Research has indicated that MTCH2 could act as a pro-cancerous gene in various cancers. MTCH2 is overexpressed in glioma, which is linked to a poor prognosis [33]. Silencing MTCH2 disrupted mitochondrial function, caused oxidative damage, impaired cell migration and invasion, inhibited the pro-survival Akt signaling pathway, and heightened sensitivity to temozolomide in glioma cells [33]. MTCH2 expression is also elevated in breast cancer, promoting cell proliferation and advancing cell cycle progression via the PI3K-Akt pathway [34]. Furthermore, a study by Li et al., discovered that apolipoprotein C1 accelerates osteosarcoma progression by interacting with MTCH2 [35].

The findings of this study indicated that MTCH2 may serve as a novel and critical therapeutic target for NSCLC. Bioinformatic analysis revealed that MTCH2 overexpression in NSCLC tissues is associated with poor prognosis and other key clinical parameters. Single-cell sequencing data also showed elevated MTCH2 levels in NSCLC cancer cells within tumors. Furthermore, MTCH2 is upregulated in both locally treated NSCLC tissues and various primary and established human NSCLC cells. In NSCLC cells, silencing MTCH2 through targeted shRNA or KO using the CRISPR/Cas9 method significantly impaired cell viability, proliferation, and migration, while promoting apoptosis. In contrast, ectopic overexpression of MTCH2 in primary human NSCLC cells enhanced cell proliferation and accelerated migration. In vivo, intratumoral injection of MTCH2 shRNA aav markedly inhibited the growth of subcutaneous xenografts of primary NSCLC cells in nude mice.

Recent studies have demonstrated that inhibition, silencing, or KO of several mitochondrial components, including POLRMT, ADCK2, TNF receptor-associated protein 1 (TRAP1) and YME1L, led to mitochondrial dysfunction, resulting in reduced energy production, increased ROS production, oxidative injury, and apoptosis in NSCLC cells [24–27]. Conversely, ectopic overexpression of these components enhanced mitochondrial function and promoted NSCLC cell growth [24–26]. Our findings indicate that MTCH2 is essential for maintaining mitochondrial hyper-function in NSCLC cells. Knockdown or KO of MTCH2 significantly impaired mitochondrial function, evidenced by reduced mitochondrial respiration, decreased complex I activity, lower ATP levels, lower mitochondrial membrane potential (mitochondrial depolarization), and increased ROS production. In contrast, ectopic overexpression of MTCH2 in primary human NSCLC cells enhanced mitochondrial complex I activity and ATP production. In MTCH2-silenced NSCLC xenograft tissues, we observed decreased complex I activity, ATP content, and GSH/GSSG ratio, alongside increased TBAR activity. These results suggest that maintaining mitochondrial hyper-function is a key mechanism by which MTCH2 drives NSCLC cell growth.

While the respiratory chain complexes are in the inner mitochondrial membrane, MTCH2, an outer membrane protein, is essential for mitochondrial dynamics and function [31]. Silencing of MTCH2 impairs various aspects of of mitochondrial architecture and functions, including impaired mitochondrial fusion [49], enlarged mitochondrial size [50], reduced mitochondrial motility [51], disrupted mitochondrial permeability transition [52], decreased ATP production [53], elevated oxidative stress [33, 50], and altered apoptosis [30, 33, 53]. In addition, MTCH2 was both required and sufficient to mediate the insertion of a wide range of mitochondrial proteins into the mitochondrial outer membrane [30], these proteins are important for mitochondrial respiration [30]. For example, MTCH2-dependent CYB5B [30] acts as an electron carrier in the mitochondrial electron transport chain, facilitating electron transfer crucial for ATP production through oxidative phosphorylation [54–56]. Additionally, FUNDC1, another protein dependent on MTCH2 [30], enhances mitochondrial respiration by promoting mitophagy and regulating dynamics, especially under hypoxic conditions [57, 58]. Importantly, we showed that mitochondrial expression of MTCH2 and FUNDC1 was significantly reduced in MTCH2-silenced NSCLC cells. Therefore, the role of MTCH2 extends beyond the outer mitochondrial membrane, as it is integral to maintaining mitochondrial architecture and function, which therefore influences the activity of the respiratory chain. Collectively, these findings indicate that MTCH2 overexpression drives NSCLC cell growth by maintaining elevated mitochondrial activity, suggesting MTCH2 as a potential therapeutic target for NSCLC.

## Supplementary information

Figure S1

## Data Availability

All data are available upon reasonable request.

## References

[1] Siegel RL, Miller KD, Jemal A. Cancer statistics, 2020. CA Cancer J Clin. 2020;70:7–30. 31912902 10.3322/caac.21590

[2] Siegel RL, Miller KD, Jemal A. Cancer statistics, 2019. CA Cancer J Clin. 2019;69:7–34. 30620402 10.3322/caac.21551

[3] Thai AA, Solomon BJ, Sequist LV, Gainor JF, Heist RS. Lung cancer. Lancet. 2021;398:535–54. 34273294 10.1016/S0140-6736(21)00312-3

[4] Arbyn M, Weiderpass E, Bruni L, de Sanjose S, Saraiya M, Ferlay J, et al. Estimates of incidence and mortality of cervical cancer in 2018: a worldwide analysis. Lancet Glob Health. 2020;8:e191–e203. 31812369 10.1016/S2214-109X(19)30482-6 PMC7025157

[5] Hirsch FR, Scagliotti GV, Mulshine JL, Kwon R, Curran WJ Jr, Wu YL, et al. Lung cancer: current therapies and new targeted treatments. Lancet. 2017;389:299–311. 27574741 10.1016/S0140-6736(16)30958-8

[6] Hiley CT, Le Quesne J, Santis G, Sharpe R, de Castro DG, Middleton G, et al. Challenges in molecular testing in non-small-cell lung cancer patients with advanced disease. Lancet. 2016;388:1002–11. 27598680 10.1016/S0140-6736(16)31340-X

[7] Arbour KC, Riely GJ. Systemic Therapy for Locally Advanced and Metastatic Non-Small Cell Lung Cancer: A Review. JAMA. 2019;322:764–74. 31454018 10.1001/jama.2019.11058

[8] Huang CY, Ju DT, Chang CF, Muralidhar Reddy P, Velmurugan BK. A review on the effects of current chemotherapy drugs and natural agents in treating non-small cell lung cancer. Biomedicine. 2017;7:23. 29130448 10.1051/bmdcn/2017070423 PMC5682982

[9] Vestergaard HH, Christensen MR, Lassen UN. A systematic review of targeted agents for non-small cell lung cancer. Acta Oncol. 2018;57:176–86. 29172833 10.1080/0284186X.2017.1404634

[10] Herbst RS, Morgensztern D, Boshoff C. The biology and management of non-small cell lung cancer. Nature. 2018;553:446–54. 29364287 10.1038/nature25183

[11] Gridelli C, Rossi A, Carbone DP, Guarize J, Karachaliou N, Mok T, et al. Non-small-cell lung cancer. Nat Rev Dis Primers. 2015;1:15009. 27188576 10.1038/nrdp.2015.9

[12] Xu P, Jiang L, Yang Y, Wu M, Liu B, Shi Y, et al. PAQR4 promotes chemoresistance in non-small cell lung cancer through inhibiting Nrf2 protein degradation. Theranostics. 2020;10:3767–78. 32206121 10.7150/thno.43142 PMC7069097

[13] Li F, Li X, Li Z, Ji W, Lu S, Xia W. betaKlotho is identified as a target for theranostics in non-small cell lung cancer. Theranostics. 2019;9:7474–89. 31695781 10.7150/thno.35582 PMC6831461

[14] Imyanitov EN, Iyevleva AG, Levchenko EV. Molecular testing and targeted therapy for non-small cell lung cancer: Current status and perspectives. Crit Rev Oncol Hematol. 2021;157:103194. 33316418 10.1016/j.critrevonc.2020.103194

[15] Fumarola C, Bonelli MA, Petronini PG, Alfieri RR. Targeting PI3K/AKT/mTOR pathway in non small cell lung cancer. Biochem Pharmacol. 2014;90:197–207. 24863259 10.1016/j.bcp.2014.05.011

[16] Forde PM, Brahmer JR, Kelly RJ. New strategies in lung cancer: epigenetic therapy for non-small cell lung cancer. Clin Cancer Res. 2014;20:2244–8. 24644000 10.1158/1078-0432.CCR-13-2088 PMC4325981

[17] Jonna S, Subramaniam DS. Molecular diagnostics and targeted therapies in non-small cell lung cancer (NSCLC): an update. Discov Med. 2019;27:167–70. 31095926

[18] Heavey S, O’Byrne KJ, Gately K. Strategies for co-targeting the PI3K/AKT/mTOR pathway in NSCLC. Cancer Treat Rev. 2014;40:445–56. 24055012 10.1016/j.ctrv.2013.08.006

[19] Porporato PE, Filigheddu N, Bravo-San Pedro JM, Kroemer G, Galluzzi L. Mitochondrial metabolism and cancer. Cell Research. 2018;28:265–80. 29219147 10.1038/cr.2017.155 PMC5835768

[20] Burke PJ. Mitochondria, Bioenergetics and Apoptosis in Cancer. Trends Cancer. 2017;3:857–70. 29198441 10.1016/j.trecan.2017.10.006 PMC5957506

[21] Vyas S, Zaganjor E, Haigis MC. Mitochondria and Cancer. Cell. 2016;166:555–66. 27471965 10.1016/j.cell.2016.07.002 PMC5036969

[22] Weinberg SE, Chandel NS. Targeting mitochondria metabolism for cancer therapy. Nat Chem Biol. 2015;11:9–15. 25517383 10.1038/nchembio.1712 PMC4340667

[23] Viale A, Corti D, Draetta GF. Tumors and mitochondrial respiration: a neglected connection. Cancer Res. 2015;75:3685–6. 26374463 10.1158/0008-5472.CAN-15-0491

[24] Xia Y, He C, Hu Z, Wu Z, Hui Y, Liu YY, et al. The mitochondrial protein YME1 Like 1 is important for non-small cell lung cancer cell growth. Int J Biol Sci. 2023;19:1778–90. 37063426 10.7150/ijbs.82217 PMC10092760

[25] Zhang JZ, Liu J, Xu YX, Pu WY, Shen MJ, Jiang KQ, et al. Identification of the mitochondrial protein ADCK2 as a therapeutic oncotarget of NSCLC. Int J Biol Sci. 2022;18:6163–75. 36439873 10.7150/ijbs.78354 PMC9682539

[26] Zhou T, Sang YH, Cai S, Xu C, Shi MH. The requirement of mitochondrial RNA polymerase for non-small cell lung cancer cell growth. Cell Death Dis. 2021;12:751. 34326320 10.1038/s41419-021-04039-2 PMC8322058

[27] Agorreta J, Hu J, Liu D, Delia D, Turley H, Ferguson DJ, et al. TRAP1 regulates proliferation, mitochondrial function, and has prognostic significance in NSCLC. Mol Cancer Res. 2014;12:660–9. 24567527 10.1158/1541-7786.MCR-13-0481 PMC5207305

[28] Lennon FE, Salgia R. Mitochondrial dynamics: biology and therapy in lung cancer. Expert Opin Investig Drugs. 2014;23:675–92. 24654596 10.1517/13543784.2014.899350

[29] Roberts ER, Thomas KJ. The role of mitochondria in the development and progression of lung cancer. Comput Struct Biotechnol J. 2013;6:e201303019. 24688727 10.5936/csbj.201303019 PMC3962144

[30] Guna A, Stevens TA, Inglis AJ, Replogle JM, Esantsi TK, Muthukumar G, et al. MTCH2 is a mitochondrial outer membrane protein insertase. Science. 2022;378:317–22. 36264797 10.1126/science.add1856 PMC9674023

[31] Peng X, Yang Y, Hou R, Zhang L, Shen C, Yang X, et al. MTCH2 in Metabolic Diseases, Neurodegenerative Diseases, Cancers, Embryonic Development and Reproduction. Drug Des Devel Ther. 2024;18:2203–13. 38882047 10.2147/DDDT.S460448 PMC11180440

[32] Zaltsman Y, Shachnai L, Yivgi-Ohana N, Schwarz M, Maryanovich M, Houtkooper RH, et al. MTCH2/MIMP is a major facilitator of tBID recruitment to mitochondria. Nat Cell Biol. 2010;12:553–62. 20436477 10.1038/ncb2057 PMC4070879

[33] Yuan Q, Yang W, Zhang S, Li T, Zuo M, Zhou X, et al. Inhibition of mitochondrial carrier homolog 2 (MTCH2) suppresses tumor invasion and enhances sensitivity to temozolomide in malignant glioma. Mol Med. 2021;27:7. 33509092 10.1186/s10020-020-00261-4 PMC7842075

[34] Jiang W, Miao Y, Xing X, Liu S, Xing W, Qian F. MTCH2 stimulates cellular proliferation and cycles via PI3K/Akt pathway in breast cancer. Heliyon. 2024;10:e28172. 38560664 10.1016/j.heliyon.2024.e28172 PMC10979243

[35] Li R, He H, He X. APOC1 promotes the progression of osteosarcoma by binding to MTCH2. Exp Ther Med. 2023;25:163. 36911382 10.3892/etm.2023.11862 PMC9996334

[36] Chen TF, Hao HF, Zhang Y, Chen XY, Zhao HS, Yang R, et al. HBO1 induces histone acetylation and is important for non-small cell lung cancer cell growth. Int J Biol Sci. 2022;18:3313–23. 35637972 10.7150/ijbs.72526 PMC9134900

[37] Xia YC, Zha JH, Sang YH, Yin H, Xu GQ, Zhen J, et al. AMPK activation by ASP4132 inhibits non-small cell lung cancer cell growth. Cell Death Dis. 2021;12:365. 33824293 10.1038/s41419-021-03655-2 PMC8024326

[38] Chen G, Mo S, Yuan D. Upregulation Mitochondrial Carrier 1 (MTCH1) Is Associated with Cell Proliferation, Invasion, and Migration of Liver Hepatocellular Carcinoma. Biomed Res Int. 2021;2021:9911784. 34195286 10.1155/2021/9911784 PMC8203356

[39] Cheng F, Huang H, Yin S, Liu JS, Sun P. Expression and functional implications of YME1L in nasopharyngeal carcinoma. Cell Death Dis. 2024;15:423. 38890304 10.1038/s41419-024-06811-6 PMC11189534

[40] Ding M, Shi R, Cheng S, Li M, De D, Liu C, et al. Mfn2-mediated mitochondrial fusion alleviates doxorubicin-induced cardiotoxicity with enhancing its anticancer activity through metabolic switch. Redox Biol. 2022;52:102311. 35413642 10.1016/j.redox.2022.102311 PMC9006862

[41] Prazanowska KH, Lim SB. An integrated single-cell transcriptomic dataset for non-small cell lung cancer. Sci Data. 2023;10:167. 36973297 10.1038/s41597-023-02074-6 PMC10042991

[42] Kim N, Kim HK, Lee K, Hong Y, Cho JH, Choi JW, et al. Single-cell RNA sequencing demonstrates the molecular and cellular reprogramming of metastatic lung adenocarcinoma. Nat Commun. 2020;11:2285. 32385277 10.1038/s41467-020-16164-1 PMC7210975

[43] Huang TW, Lin KF, Lee CH, Chang H, Lee SC, Shieh YS. The role of Thyroid Transcription Factor-1 and Tumor differentiation in Resected Lung Adenocarcinoma. Sci Rep. 2017;7:14222. 29079814 10.1038/s41598-017-14651-y PMC5660159

[44] Turner BM, Cagle PT, Sainz IM, Fukuoka J, Shen SS, Jagirdar J. Napsin A, a new marker for lung adenocarcinoma, is complementary and more sensitive and specific than thyroid transcription factor 1 in the differential diagnosis of primary pulmonary carcinoma: evaluation of 1674 cases by tissue microarray. Arch Pathol Lab Med. 2012;136:163–71. 22288963 10.5858/arpa.2011-0320-OA

[45] Stoll LM, Johnson MW, Gabrielson E, Askin F, Clark DP, Li QK. The utility of napsin-A in the identification of primary and metastatic lung adenocarcinoma among cytologically poorly differentiated carcinomas. Cancer Cytopathol. 2010;118:441–9. 20830690 10.1002/cncy.20108

[46] Schrank Z, Chhabra G, Lin L, Iderzorig T, Osude C, Khan N, et al. Current Molecular-Targeted Therapies in NSCLC and Their Mechanism of Resistance. Cancers. 2018;10:224. 10.3390/cancers10070224 PMC6071023 29973561

[47] Tang S, Qin C, Hu H, Liu T, He Y, Guo H, et al. Immune Checkpoint Inhibitors in Non-Small Cell Lung Cancer: Progress, Challenges, and Prospects. Cells. 2022;11:320. 10.3390/cells11030320 PMC8834198 35159131

[48] Zhou F, Qiao M, Zhou C. The cutting-edge progress of immune-checkpoint blockade in lung cancer. Cell Mol Immunol. 2021;18:279–93. 33177696 10.1038/s41423-020-00577-5 PMC8027847

[49] Bahat A, Goldman A, Zaltsman Y, Khan DH, Halperin C, Amzallag E, et al. MTCH2-mediated mitochondrial fusion drives exit from naive pluripotency in embryonic stem cells. Nat Commun. 2018;9:5132. 30510213 10.1038/s41467-018-07519-w PMC6277412

[50] Maryanovich M, Zaltsman Y, Ruggiero A, Goldman A, Shachnai L, Zaidman SL, et al. An MTCH2 pathway repressing mitochondria metabolism regulates haematopoietic stem cell fate. Nat Commun. 2015;6:7901. 26219591 10.1038/ncomms8901

[51] Ruggiero A, Aloni E, Korkotian E, Zaltsman Y, Oni-Biton E, Kuperman Y, et al. Loss of forebrain MTCH2 decreases mitochondria motility and calcium handling and impairs hippocampal-dependent cognitive functions. Sci Rep. 2017;7:44401. 28276496 10.1038/srep44401 PMC5343590

[52] Guo L. Mitochondrial permeability transition mediated by MTCH2 and F-ATP synthase contributes to ferroptosis defense. FEBS Lett. 2024. 10.1002/1873-3468.15008 39227319

[53] Sun G, Song Y, Li C, Sun B, Li C, Sun J, et al. MTCH2 promotes the malignant progression of ovarian cancer through the upregulation of AIMP2 expression levels, mitochondrial dysfunction and by mediating energy metabolism. Oncol Lett. 2024;28:492. 39185493 10.3892/ol.2024.14625 PMC11342418

[54] Hall R, Yuan S, Wood K, Katona M, Straub AC. Cytochrome b5 reductases: Redox regulators of cell homeostasis. J Biol Chem. 2022;298:102654. 36441026 10.1016/j.jbc.2022.102654 PMC9706631

[55] Rodriguez-Maranon MJ, Qiu F, Stark RE, White SP, Zhang X, Foundling SI, et al. 13C NMR spectroscopic and X-ray crystallographic study of the role played by mitochondrial cytochrome b5 heme propionates in the electrostatic binding to cytochrome c. Biochemistry. 1996;35:16378–90. 8973214 10.1021/bi961895o

[56] Durley RC, Mathews FS. Refinement and structural analysis of bovine cytochrome b5 at 1.5 A resolution. Acta Crystallogr D Biol Crystallogr. 1996;52:65–76. 15299727 10.1107/S0907444995007827

[57] Bai X, Zhang Z, Li X, Yang Y, Ding S. FUNDC1: An Emerging Mitochondrial and MAMs Protein for Mitochondrial Quality Control in Heart Diseases. Int J Mol Sci. 2023;24:9151. 10.3390/ijms24119151 PMC10252584 37298100

[58] Liu H, Zang C, Yuan F, Ju C, Shang M, Ning J, et al. The role of FUNDC1 in mitophagy, mitochondrial dynamics and human diseases. Biochem Pharmacol. 2022;197:114891. 34968482 10.1016/j.bcp.2021.114891

