# Supplementary figures and images for: Mitochondrial carrier homolog 2 is important for mitochondrial functionality and non-small cell lung cancer cell growth
Source: Cell Death Dis. 2025 Feb 13;16(1):95. doi: 10.1038/s41419-025-07419-0 (PMC11825924; doi:10.1038/s41419-025-07419-0)

Figure S1. The uncropped blotting images of the study.

Figure 3.

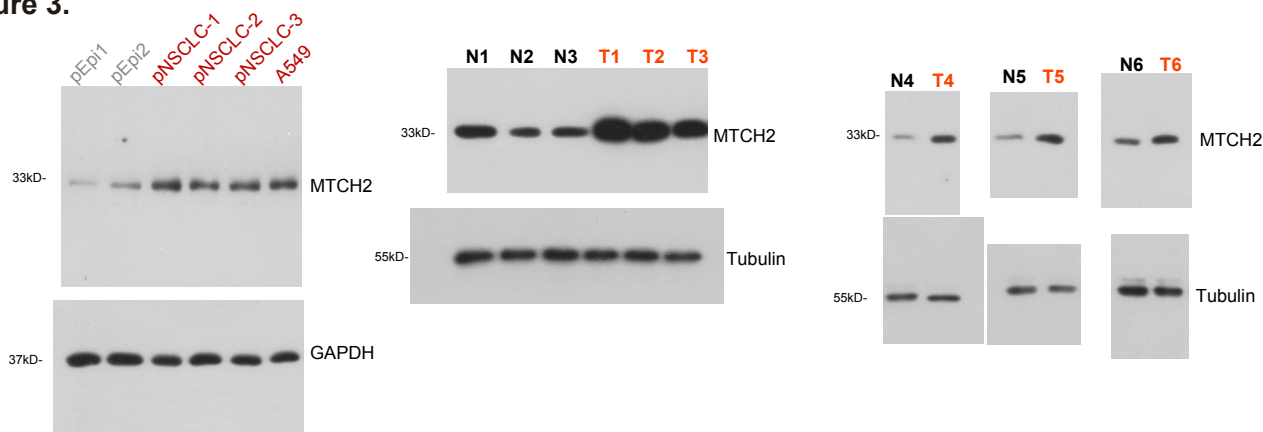

Figure 4.

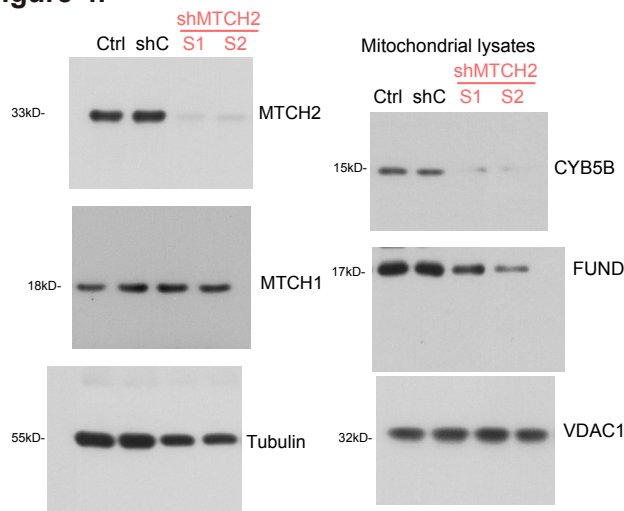

Figure 8.

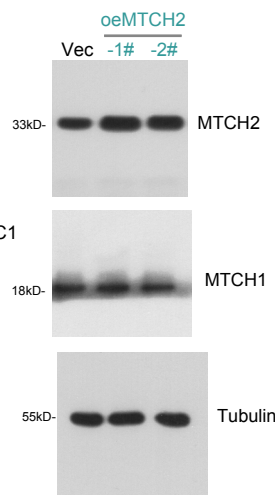

Figure 7.

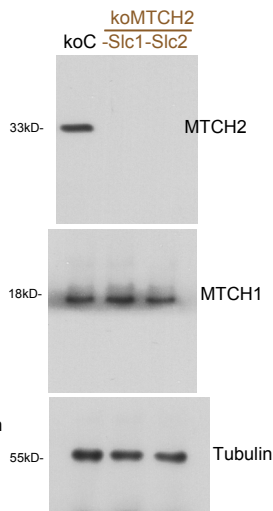

Figure 5.

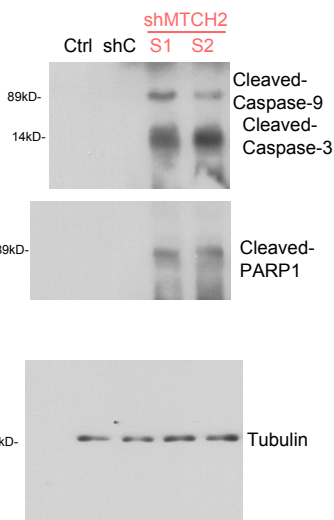

Figure 9.

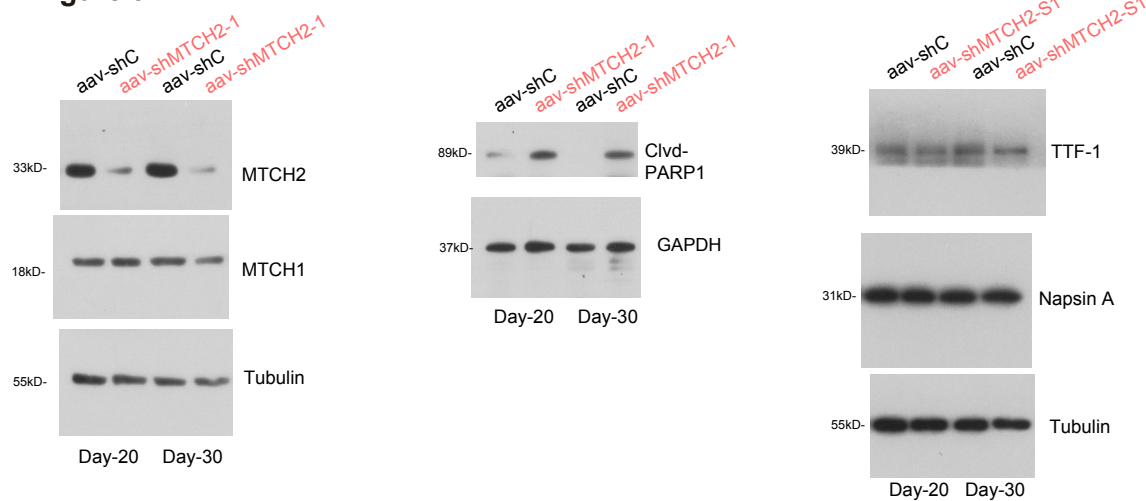

Supplement: Supplementary file 1 — Figure S1 [file 41419_2025_7419_MOESM1_ESM.pdf]
